# Supplementary material for: Calcium supplementation to prevent pre-eclampsia: protocol for an individual participant data meta-analysis, network meta-analysis and health economic evaluation
Source: BMJ Open. 2023 May 11;13(5):e065538. doi: 10.1136/bmjopen-2022-065538 (PMC10186423; doi:10.1136/bmjopen-2022-065538)
Supplement: Supplementary data [file bmjopen-2022-065538supp001.pdf]

Appendix: Details of search strategies used in IPD meta-analysis on calcium supplementation to prevent pre-eclampsia in low- income and middle-income countries

1. Ovid MEDLINE

1 exp Pregnancy/
2 Pregnant Women/
3 (pregnan$ or gestation$ or antenatal$ or ante-natal$ or prenatal$ or pre-natal$ or post-conception$ or postconception$).ti,ab,kf.
4 or/1-3
5 Calcium/
6 Calcium, Dietary/
7 calcium.ti,ab,kf.
8 or/5-7
9 4 and 8
10 randomized controlled trial.pt.
11 controlled clinical trial.pt.
12 randomized.ab.
13 placebo.ab.
14 Clinical Trials as Topic/
15 randomly.ab.
16 trial.ti.
17 or/10-16
18 9 and 17
19 exp Animals/ not Humans/
20 18 not 19
21 (news or editorial or case reports).pt. or case report.ti.
22 20 not 21
23 remove duplicates from 22

2. Cochrane Central Register of Controlled Trials

#1 [mh Pregnancy]

#2 [mh ^"Pregnant Women"]

#3 (pregnan* or wom* or gestation* or antenatal* or prenatal* or postconception* or ante NEXT natal* or pre NEXT natal* or post NEXT conception*)

#4 #1 OR #2 OR #3

#5 [mh ^"Calcium, Dietary"]

#6 [mh ^calcium]

#7 calcium

#8 #5 OR #6 OR #7

#9 #4 AND #8 in Trials

3. WHO International Clinical Trials Platform

pregnancy AND calcium OR wom* AND calcium OR gestation* AND calcium OR antenatal* AND calcium OR prenatal* AND calcium OR postconception* AND calcium OR antenatal* AND calcium OR prenatal* AND calcium OR postconception* AND calcium

4. ClinicalTrials.gov

calcium | Interventional Studies | pregnancy OR pregnant OR woman OR women OR gestation OR antenatal OR prenatal OR postconception OR ante-natal OR pre-natal OR post-conception | Studies with Female Participants

5. SCOPUS

((TITLE-ABS-KEY (pregnan* OR wom* OR gestation* OR antenatal* OR "ante-natal*" OR prenatal OR "pre-natal*" OR "post-conception*" OR postconception*) AND TITLE-ABS-KEY (calcium) AND TITLE-ABS-KEY (trial* OR random* OR "clinical stud*" OR "controlled stud*"))) AND NOT ((KEY (animal*) OR TITLE (rat OR rats OR mice OR mouse OR hamster OR hamsters OR bovine OR sheep OR dog OR dogs OR cat OR cats OR rabbit OR rabbits OR calf OR calves OR cow OR cows OR pig OR pigs OR swine OR porcine)) AND NOT KEY (human*)) AND NOT INDEX (medline)

6. CINAHL

S1 (MH "Pregnancy+”)
S2 (MH “Expectant Mothers”)
S3 TI (pregnan* or gestation* or antenatal* or prenatal* or postconception* or ante W3 natal* or pre W3 natal* or post W3 conception*) OR AB (pregnan* or gestation* or antenatal* or prenatal* or postconception* or ante W3 natal* or pre W3 natal* or post W3 conception*)
S4 S1 OR S2 OR S3
S5 (MH "Calcium")
S6 (MH "Calcium, Dietary")
S7 TI calcium OR AB calcium
S8 S5 OR S6 OR S7
S9 S4 AND S8
S10 (MH “randomized controlled trials”)
S11 (MH double-blind studies)
S12 (MH “single-blind studies”)
S13 (MH “random assignment”)
S14 (MH “pretest-posttest design”)
S15 (MH “cluster sample”)
S16 TI (randomised OR randomized)
S17 AB (random*)

Rocha T, *et al. BMJ Open* 2023; 13:e065538. doi: 10.1136/bmjopen-2022-065538

S18 TI (trial)
S19 MH (“sample size”) AND AB (assigned OR allocated OR control)
S20 MH (placebos)
S21 PT (“randomized controlled trial”)
S22 AB (control W5 group)
S23 MH (crossover design) OR MH (comparative studies)
S24 AB (cluster W3 RCT)
S25 S10 OR S11 OR S12 OR S13 OR S14 OR S15 OR S16 OR S17 OR S18 OR S19 OR S20 OR S21 OR S22 OR S23 OR S24
S26 S9 AND S25
S27 (MH Animals+) OR (MH “Animal Studies”) OR TI (animal model*)
S28 MH (Human)
S29 S27 NOT S28
S30 S26 NOT S29

7. PubMed

"(((calcium supplement*) OR (“calcium carbonate”) OR (“calcium gluconate”) OR (“calcium acetate”) OR (“calcium citrate”) OR (“calcium lactate”) OR (“calcium”)) AND ((“Pregnant Women”[Mesh]) OR (“Pregnancy”[Mesh]) OR (“pregnancy”) OR (“pregnant”) OR (“pregnancies”)) AND ((random) OR (randomised) OR (randomized)) AND (trial)"

8. EMBASE, CINHAL, AMED, and LILACS.

"(calcium) AND (pregnan*) AND ((random) OR (randomised) OR (randomized)) AND (trial)”
