# Supplementary material for: Calcium supplementation to prevent pre-eclampsia: protocol for an individual participant data meta-analysis, network meta-analysis and health economic evaluation
Source: BMJ Open. 2023 May 11;13(5):e065538. doi: 10.1136/bmjopen-2022-065538 (PMC10186423; doi:10.1136/bmjopen-2022-065538)
Supplement: Author's manuscript [file bmjopen-2022-065538.draft_revisions.pdf]

BMJ Open is committed to open peer review. As part of this commitment we make the peer review history of every article we publish publicly available.

When an article is published we post the peer reviewers' comments and the authors' responses online. We also post the versions of the paper that were used during peer review. These are the versions that the peer review comments apply to.

The versions of the paper that follow are the versions that were submitted during the peer review process. They are not the versions of record or the final published versions. They should not be cited or distributed as the published version of this manuscript.

BMJ Open is an open access journal and the full, final, typeset and author-corrected version of record of the manuscript is available on our site with no access controls, subscription charges or pay-per-view fees (http://bmjopen.bmj.com).

If you have any questions on BMJ Open's open peer review process please email info.bmjopen@bmj.com

BMJ Open

Study protocol: Calcium supplementation to prevent pre-eclampsia in low- and middle-income countries – individual participant data (IPD) meta-analysis, network meta-analysis, and health economic evaluation

Journal:	*BMJ Open*
Manuscript ID	bmjopen-2022-065538
Article Type:	Protocol
Date Submitted by the Author:	13-Jun-2022
Complete List of Authors:	Rocha, Thaís; University of Birmingham, WHO Collaborating Centre for Global Women's Health, Institute of Metabolism and Systems Research<br>Allotey, John; University of Birmingham, WHO Collaborating Centre for Global Women's Health, Institute of Metabolism and Systems Research<br>Palacios , Alfredo ; Institute for Clinical Effectiveness and Health Policy, Health Economics<br>Vogel, J; Burnet Institute, Maternal, Child and Adolescent Health Program<br>Smits, Luc; Maastricht University, Department of Epidemiology, Care and Public Health Research Institute<br>Carroli, Guillermo; Centro Rosarino de Estudios Perinatales (CREP)<br>Mistry, Hema; University of Warwick, Warwick Evidence<br>Young, Taryn; Stellenbosch University, Centre for Evidence-based Health Care, Faculty of Medicine and Health Sciences; South African Medical Research Council, South African Cochrane Centre<br>Qureshi , Zahida ; University of Nairobi Department of Obstetrics and Gynecology<br>Cormick, G; Institute for Clinical Effectiveness and Health Policy<br>Snell, Kym IE; Keele University<br>Abalos, E; Centro Rosarino de Estudios Perinatales, Moreno, Argentina<br>Pena-Rosas, Juan-Pablo; World Health Organization, Reproductive Health and Research<br>Khan , Khalid; University of Granada Faculty of Medicine, Public Health<br>Larbi, Koiwah Koi; Action on Preeclampsia (APEC)<br>Thorson, Anna; World Health Organization, Reproductive Health and Research<br>Singata-Madliki, Mandisa; East London Hospital Complex, Effective Care Research Unit (ECRU)<br>Hofmeyr, G Justus ; Frere Hospital, Obstetrics and Gynaecology<br>Bohren, Meghan ; University of Melbourne School of Population and Global Health<br>Riley, Richard; Keele University<br>Betran, Ana Pilar; World Health Organization, Reproductive Health and Research<br>Thangaratinam, Shakila; University of Birmingham College of Medical and Dental Sciences, WHO Collaborating Centre for Global Women's Health, Institute of Metabolism and Systems Research; Birmingham Women's and Children's Hospitals NHS Foundation Trust

Keywords:	HEALTH ECONOMICS, Maternal medicine < OBSTETRICS, PUBLIC HEALTH

SCHOLARONE™
Manuscripts

Study protocol: Calcium supplementation to prevent pre-eclampsia in low- and middle-income countries – individual participant data (IPD) meta-analysis, network meta-analysis, and health economic evaluation

Thaís Rocha,[1] John Allotey,[1] Alfredo Palacios,[2] Joshua P Vogel,[3] Luc Smits,[4] Guillermo Carroli,[5] Hema Mistry,[6] Taryn Young,[7] Zahida Qureshi,[8] Gabriela Cormick,[9] Kym IE Snell,[10] Edgardo Abalos,[5] Juan Pablo Pena-Rosas,[11] Khalid Khan,[12] Koiwah Koi Larbi,[13] Ana Thorson,[11] Mandisa S Madliki,[14] Justus Hofmeyr,[14] Meghan A Bohren,[15] Richard D Riley,[10] Ana Pilar Betrán,[11] Shakila Thangaratinam[1,16] on behalf of the International Calcium in Pregnancy (i-CIP) Collaborative Network

AFFILIATION:

1. WHO Collaborating Centre for Global Women's Health, Institute of Metabolism and Systems Research, University of Birmingham, Birmingham, United Kingdom
2. Institute for Clinical Effectiveness and Health Policy (IECS), Buenos Aires, Argentina
3. Maternal, Child and Adolescent Health Program, Burnet Institute, Melbourne, Australia
4. Department of Epidemiology, Care and Public Health Research Institute, Maastricht University, Maastricht, the Netherlands
5. Centro Rosarino de Estudios Perinatales (CREP), Rosario, Argentina
6. Clinical Trials Unit, Warwick Medical School, University of Warwick, Coventry, United Kingdom
7. Centre for Evidence-based Health Care, Division of Epidemiology and Biostatistics, Department of Global Health, Faculty of Medicine and Health Sciences, Stellenbosch University, Stellenbosch, South Africa

For peer review only - http://bmjopen.bmj.com/site/about/guidelines.xhtml

8. The University of Nairobi, Nairobi, Kenya
9. Department of Mother and Child Health Research, Institute for Clinical Effectiveness and Health Policy (IECS-CONICET), Buenos Aires, Argentina
10. Centre for Prognosis Research, School of Medicine, Keele University, Staffordshire, United Kingdom
11. UNDP, UNFPA, UNICEF, WHO, World Bank Special Programme of Research, Development and Research Training in Human Reproduction (HRP), Department of Reproductive Health and Research, World Health Organization, Geneva, Switzerland.
12. The University of Granada, Granada, Spain
13. Action on preeclampsia Charity (APEC), Ghana
14. Effective Care Research Unit, University of Witwatersrand, South Africa
15. Gender and Women's Health Unit, Centre for Health Equity, School of Population and Global Health, University of Melbourne, Melbourne, Australia
16. Birmingham Women's and Children's NHS Foundation Trust, Birmingham, UK

CORRESPONDING AUTHOR:

Thais Rocha

Institute of Metabolism and Systems Research, IBR Level 3

University of Birmingham, Birmingham, United Kingdom, B15 2TT

t.rocha@bham.ac.uk

WORD COUNT: 3,714 words

For peer review only

For peer review only - http://bmjopen.bmj.com/site/about/guidelines.xhtml

KEYWORDS: pre-eclampsia, calcium supplementation, pregnancy, IPD meta-analysis, economic evaluation, network meta-analysis

ABSTRACT

Introduction

Low dietary calcium intake is a risk factor for pre-eclampsia, a major contributor to maternal and perinatal mortality and morbidity worldwide. Calcium supplementation can prevent pre-eclampsia in women with low dietary calcium. However, the optimal dose and timing of calcium supplementation are not known. We plan to undertake an individual participant data (IPD) meta-analysis of randomised trials to determine the effects of various calcium supplementation regimens in preventing pre-eclampsia and its complications and rank these by effectiveness. We also aim to evaluate the cost-effectiveness of calcium supplementation to prevent pre-eclampsia.

Methods and analysis

We will identify randomised trials on calcium supplementation before and during pregnancy by searching major electronic databases from inception to February 2022. Primary researchers of the identified trials will be invited to join the International Calcium in Pregnancy (i-CIP) Collaborative Network and share their IPD. We will check each study's IPD for consistency with the original authors before standardising and harmonising the data. We will perform a series of one- and two-stage IPD random-effect meta-analyses to obtain the summary intervention effects on pre-eclampsia with 95% confidence intervals and summary treatment-covariate interactions (maternal risk status, dietary intake, timing of intervention, daily dose of calcium prescribed, and total intake of calcium). Heterogeneity will be summarised using tau-squared, I-squared and 95% prediction intervals for effect in a new study. Minor study effects (potential publication bias) will

For peer review only - http://bmjopen.bmj.com/site/about/guidelines.xhtml

be investigated using funnel plots. A decision-analytic model for use in low- and middle-income countries will assess the cost-effectiveness of calcium supplementation to prevent pre-eclampsia.

Ethics and dissemination

No ethical approvals are required. We will store the data in a secure repository in an anonymised format. The results will be published in peer-reviewed journals.

PROSPERO registration number CRD42021231276.

ARTICLE SUMMARY

Strengths and limitations of this study

- The IPD approach will increase our ability to explore any differential treatment effect across groups. We can model how individual-level covariates (e.g., age, risk of pre-eclampsia) interact with treatment effect within the same trial and possibly explain variability in patient outcomes.
- By analysing data on the actual amount of calcium taken by an individual woman and her adherence to the prescribed regimen, we can explore the doses and frequencies of the clinical benefits of calcium supplementation. This can inform future clinical recommendations on calcium dose and adherence requirements and strategies for food or water fortification programmes.
- The health-economic analysis will assist policymakers, healthcare managers and other healthcare service providers in informing decisions regarding the ongoing use or future calcium supplementation strategies to prevent pre-eclampsia based on the efficiency principle.

For peer review only - http://bmjopen.bmj.com/site/about/guidelines.xhtml

- Limitations include potential individual participant data unavailability (e.g., lack of specific data such as gestational age and adherence) and low quality of trial data.

BACKGROUND

Pre-eclampsia is a pregnancy-specific condition characterised by raised blood pressure and protein in the urine. It is a major cause of maternal and perinatal mortality and morbidity worldwide, contributing to 76,000 maternal and half a million perinatal deaths each year; - 99% of these are from low-and-middle-income countries (LMICs).[1-3] Most maternal deaths due to pre-eclampsia are preventable. Prevention of pre-eclampsia and its complications is crucial to achieving the health-related Sustainable Development Goals (SDGs), and the World Health Organization's (WHO) Thirteenth General Programme of Work for universal health coverage.[4]

Low dietary calcium is a recognised risk factor for pre-eclampsia.[5-7] In LMICs, 80% of pregnant women have a mean calcium intake below the recommended level of 800mg/day compared with low intake in only about a quarter of pregnant women in high-income countries (HIC).[8] Calcium supplementation in pregnancy has been shown to reduce the risk of pre-eclampsia.[9] In populations with low dietary calcium intake and in those at high risk of developing pre-eclampsia, the WHO recommends 1.5–2.0g per day of oral elemental calcium supplementation during pregnancy to reduce the risk of pre-eclampsia, although there is no clear recommendation on the timing of initiation.[10]

A Cochrane review showed that high dose (≥1000mg per day) of calcium supplementation during pregnancy reduced the risk of pre-eclampsia (eight trials, 10,678 women: average RR

For peer review only - http://bmjopen.bmj.com/site/about/guidelines.xhtml

0.36, 95% CI 0.20 to 0.65; I2 = 76%). But the quality was graded low due to significant heterogeneity from variations in the underlying risk of pre-eclampsia.[9] Evidence for a low dose calcium supplement to prevent pre-eclampsia (<1000 mg/day) is limited. [9] Despite countries including calcium in their essential medicines lists, maternal mortality from hypertensive disorders in LMICs remains high.[11, 12] Optimising calcium intake to prevent preeclampsia is a priority area for the WHO.[4, 13] The 2018 WHO Guideline Development Group (GDG) highlighted research on the minimal dose and optimal commencement schedule for calcium supplementation as a high research priority.[13] It is also not known whether calcium supplementation strategies should target high-risk women only or provide calcium supplements to all pregnant and reproductive-aged women, to confer benefits and be cost-effective in preventing pre-eclampsia.

We plan to undertake an individual participant data (IPD) meta-analysis of calcium supplementation to determine the intervention effects on pre-eclampsia and its complications, assess if the effects vary according to maternal and intervention characteristics, and the cost-effectiveness of the different interventions strategies.

OBJECTIVES

Our primary objective is to determine the overall, and differential effects of calcium supplementation (according to maternal and intervention characteristics) on pre-eclampsia adjusted for co-interventions and baseline maternal calcium status, using an IPD meta-analysis.

Our secondary objectives are to:

For peer review only - http://bmjopen.bmj.com/site/about/guidelines.xhtml

- Evaluate the effects of calcium supplementation on (i) maternal outcomes such as maternal death, eclampsia, severe maternal morbidity, admission to intensive care unit, Haemolysis, Elevated Liver enzymes, Low Platelets (HELLP) syndrome; and (ii) perinatal outcomes such as stillbirth, perinatal death, neonatal death, preterm birth, low Apgar score, small for gestational age baby, and admission and length of stay in the neonatal intensive care unit.
- Produce a rank order of calcium supplementation regimens by effectiveness.
- Develop a decision-analytic model to determine the cost-effectiveness of different calcium supplementation strategies in an LMIC setting.

METHODS

Our IPD meta-analytical approach will follow existing methodological guidelines and adhere to the PRISMA-IPD reporting statement.[14] The protocol has been registered on the International Prospective Register of Systematic Reviews (PROSPERO CRD42021231276).

Patient and Public Involvement

Women with lived experience of pre-eclampsia will be involved with this work throughout and have informed the design, outcome selection and reporting.

Literature search

We will update the search of the 2018 Cochrane review [9] until February 2022 to identify new trials that have been published since the last conducted search. This will include searches in databases such as Embase, CINAHL, MEDLINE, CENTRAL, PubMed, Scopus, AMED,

For peer review only - http://bmjopen.bmj.com/site/about/guidelines.xhtml

LILACS, POPLINE, AIM, IMSEAR, ClinicalTrials.gov, and WHO International Clinical Trials Registry Platform, using search strategies adapted from the original Cochrane search. No language restrictions will be applied.

Eligibility criteria

Any clinical trial with random allocation (individual or cluster) to calcium supplementation (any dose with or without additional supplements or treatments) before or during pregnancy compared with placebo, aspirin, or routine care will be eligible for inclusion. Non-randomised trials and animal studies will be excluded.

Outcome measures

Study outcomes were informed by the WHO recommendation on calcium supplementation during pregnancy to prevent pre-eclampsia and its complications,[15] and the core outcome set for pre-eclampsia research.[16] The primary outcomes are (i) any onset pre-eclampsia and (ii) early-onset pre-eclampsia (diagnosed <34 weeks' gestation). We will use the authors' reported definition of pre-eclampsia. However, suppose the trial IPD reports relevant variables. In that case, we will redefine pre-eclampsia as high blood pressure (defined as systolic blood pressure ≥140mmHg or diastolic blood pressure ≥90mmHg after 20 weeks of pregnancy) with significant proteinuria (defined as urine protein-creatinine ratio ≥30 mg/mmol or ≥2+ on dipstick testing or ≥300 mg/24 hours or ≥500 mg per litre).

Our secondary outcomes include maternal and offspring complications such as maternal death, eclampsia, severe maternal morbidity (renal, haematological, neurological, hepatic

For peer review only - http://bmjopen.bmj.com/site/about/guidelines.xhtml

complications), admission to intensive care unit, HELLP syndrome, stillbirth, neonatal death, admission and length of stay in the neonatal intensive care unit, preterm birth, or small for gestational age (Table 1). We will undertake a subgroup analysis to explore whether the intervention effect is modified by (interacts with) maternal risk status, dietary intake, the timing of intervention, the daily dose of calcium prescribed, and total intake of calcium.

Table 1. Structured research question

**Question components**	
Population	Pregnant women and women of reproductive age who are not yet pregnant but intending to become pregnant.
Intervention	Calcium supplementation (with or without additional supplements or treatments)
Outcomes	**Primary outcome**<br>Any onset pre-eclampsia<br>Early-onset pre-eclampsia (<34 weeks' gestation)<br><br>**Secondary outcomes**<br>*Maternal outcomes*<br>Maternal death<br>Eclampsia<br>Severe maternal morbidity (renal, haematological, neurological, hepatic complications)<br>Admission to intensive care unit<br>HELLP syndrome<br><br>*Neonatal outcomes*<br>Stillbirth, neonatal death<br>Apgar score <7 after 5 minutes<br>Admission to the neonatal intensive care unit within 28 days after birth<br>Preterm birth<br>Small for gestational age baby
Design of included studies	Randomised trials

For peer review only - http://bmjopen.bmj.com/site/about/guidelines.xhtml
For peer review only

Study Selection

At least two researchers will independently select studies using a two-stage process. They will first screen the titles and abstracts of studies and then assess the full text of selected studies in detail for eligibility. Disagreement will be resolved via discussion with a third researcher. Data extraction will be done in duplicates. At the study level, extracted data will include country, setting, inclusion and exclusion criteria of participants, intervention, control, primary aim, and definition and assessment of the primary outcome.

Establishment of the International Calcium in pregnancy (i-CIP) Collaborative network

We will contact primary researchers of identified studies via email and invite them to join the collaborative network and share their IPD. To date, seven collaborators have joined the network and shared access to anonymised individual data of 16,111 women (Table 2). The network is a global effort to bring together researchers, clinicians and epidemiologists (https://www.icipnetwork.com/). A bespoke database will be set up for collaborators to share data. Authors will be allowed to share their data in any format convenient to them. We will consider all variables recorded in the original studies, even those not reported in the publications. Once deposited, the data will be converted to a standardised format, followed by the range and data consistency checking before merging and harmonising.

Table 2. List of trials current in the i-CIP network and trials that have agreed to share data (total n=17,526 individuals).

Author, Year	Country	Study population risk of PE / Start of intervention	Intervention	Comparator	Samp le size	Data already shared with the

For peer review only

							i-CIP network
Trials currently in iCIP (n=16,111 individuals, 7 trials) [data available already]							
Villar, 2006	Argentina, Egypt, India, Peru, South Africa, Vietnam	High risk, up to 20 weeks' gestation	1,500 mg calcium carbonate	Placebo	8325	Yes
Levine, 1997	United States of America	Low risk, 13-21 weeks' gestation	2,000 mg calcium carbonate	Placebo	4589	Yes
Belizán, 1991	Argentina	Any risk, 20 weeks' gestation	2,000 mg calcium carbonate	Placebo	1194	Yes
Ettinger, 2009	Mexico	Low risk, first trimester	1,200 mg calcium carbonate	Placebo	670	Yes
Goldberg, 2013	Gambia	Any risk, 18-20 weeks' gestation	1,500 mg calcium carbonate	Placebo	662	Yes
Hofmeyr, 2019	Argentina, South Africa, Zimbabwe	High risk, pre-pregnancy and up to 20 weeks' gestation	500 mg calcium carbonate	Placebo	581	Yes
Azami, 2017	Iran	High risk, > 20 weeks' gestation	800mg calcium carbonate	Multivitamin	90	Yes
Trials that agreed to share IPD (n=1,415 individuals, 7 trials) [data expected to be made available to us]							
Omotayo, 2018	Kenya	Low risk, 16-30 gestational weeks	1,500 mg calcium carbonate	1,000 mg calcium carbonate	990	No
Asemi, 2014	Iran	Low risk, 16 weeks' gestation	Multivitamin-mineral with 250 mg calcium	Multivitamin	104	No
Karamali, 2016	Iran	High risk, 24-26 weeks' gestation	1,000 mg calcium carbonate, 50,000 IU vitamin D3	Placebo	60	No
Samimi, 2016	Iran	High risk, 20 weeks' gestation	1,000 mg calcium	Placebo	60	No

For peer review only

			carbonate, 50,000 IU vitamin D3			
Souza, 2014	Brazil	High risk, 20-27 weeks' gestation	2,000 mg calcium carbonate, 100 mg aspirin	Placebo	49	No
Asemi, 2015	Iran	High risk, 27 weeks' gestation	800 mg calcium carbonate, 200 mg magnesium, 8 mg zinc, 400 IU vitamin D3	Placebo	46	No
Asemi, 2016	Iran	Low risk, 25 weeks' gestation	500 mg calcium carbonate, 200 IU vitamin D3	Placebo	46	No

Quality Assessment

The quality of the IPD from each study will be assessed independently by two researchers. We will use the revised Cochrane tool for assessing the risk of bias in randomised trials (RoB2)[17] based on published study characteristics and supplement this with information within the IPD. We will consider six items used in the Cochrane risk of bias tool: sequence generation, allocation concealment, blinding, incomplete outcome data, selective outcome reporting, and other potential sources of bias. We will conduct sensitivity analyses to examine the robustness of statistical and clinical conclusions to inform the inclusion or exclusion of trials considered to be at high risk of bias.

Data and integrity checks

We will perform integrity checks of IPD received for each trial by evaluating the integrity of randomisation and follow-up procedures and reviewing the completeness and accuracy of the

For peer review only - http://bmjopen.bmj.com/site/about/guidelines.xhtml
For peer review only

data.[18] Any inconsistencies found (missing data, extreme values, discrepancies between the trial report and the data) will be resolved with the original study authors. The study progress and discrepancies will be recorded.

Sample Size considerations

Formal sample size calculations are not usually undertaken for meta-analyses. A single trial would need 10,847 participants (80% power, 5% error) to detect the interaction odds ratio (OR) of 0.62 between low and high-risk groups, assuming calcium reduces pre-eclampsia by 20% in a low-risk group by another 30% in the high-risk population.[19] Using power calculations by simulating IPD to match aggregate data (e.g., number of participants, events, covariate distributions)[20] from studies promising their IPD so far (17,526 women) and assuming heterogeneity of 1-8% in the rates of pre-eclampsia in the low-risk group in each trial, we will have over 98% power to detect an interaction OR of 0.62 in our IPD meta-analysis.[20] Even when we additionally assume heterogeneity in the overall effect of calcium in the low-risk group from 0.6 to 0.9, the power will still be 90%, illustrating the large sample size available. We will have similar power for other covariates.

Statistical analysis

Overall effect

We will perform a series of one and two-stage IPD random-effect meta-analyses fitted using either frequentist methods (e.g., restricted maximum likelihood with confidence intervals derived using Hartung-Knapp correction) or Bayesian methods (e.g., with vague or empirically derived prior distributions). In the two-stage approach, firstly, the IPD will be analysed separately for

For peer review only - http://bmjopen.bmj.com/site/about/guidelines.xhtml

each study to obtain relevant aggregate data (e.g., a treatment effect estimates and its confidence interval for each study) for each outcome; secondly, this aggregate data will be combined (pooled) across studies using an appropriate meta-analysis model to produce relevant summary results (e.g., a weighted average of the treatment effect). The alternative one-stage approach analyses the IPD from all studies in a single step, using a statistical model (e.g., a mixed development linear, logistic or Cox regression model) that accounts for the clustering of patients within studies and potential heterogeneity between studies. When the same modelling assumptions and estimation methods are used, one-stage and two-stage approaches are similar.[21] The one-stage approach is preferable when rare events are modelled as a more exact likelihood. However, the two-stage approach allows more familiar meta-analysis techniques and graphs (e.g., forest plots). Therefore, we will perform both one-stage and two-stage methods and compare any differences.[21]

Differential effect by subgroups (treatment-covariate interactions)

For each outcome, we will examine differences in pre-defined subgroups to summarise whether the intervention effect is modified by (interacts with) maternal risk status, dietary intake, the timing of intervention, a daily dose of calcium prescribed, and total intake of calcium; this analysis will utilise only within-study information to avoid ecological bias from across study information. The one-stage analyses will be achieved by centring patient-level covariates by their mean and including the mean as an additional covariate.[22] Non-linear interactions with continuous covariates (e.g., risk status) will be examined using restricted cubic splines.[23]

IPD network meta-analysis

For peer review only - http://bmjopen.bmj.com/site/about/guidelines.xhtml

An IPD network meta-analysis will compare and rank intervention effects for the various regimens (and doses), utilising direct and indirect comparisons whilst adjusting for covariates that modify treatment effects to alleviate any inconsistency in the network.[24] The within-study correlation of multiple intervention effects from the same trial will be accounted for (if necessary). A common between-study variance is assumed for all treatment contrasts in the network. We will produce summary (pooled) effect estimates for each treatment contrast (i.e., each pair of strategies in the network) with 95% confidence intervals (CI) and the borrowing of strength statistics (to reveal the contributions of indirect evidence). Based on the results, the ranking of intervention types will be calculated using resampling methods and quantified by the probabilities of being ranked first, second, and last, together with the mean rank and the Surface Under the Cumulative Ranking curve (SUCRA). The consistency assumption will be examined for each treatment comparison with direct and indirect evidence (seen as a closed-loop within the network plot); this involves estimating the direct and indirect evidence and comparing the two.[25] The consistency assumption will also be examined across the whole network using ' design-by-treatment interaction' models, which allow an overall significance test for inconsistency. If evidence of inconsistency is found, explanations will be sought and resolved by adjusting for covariates that act as effect modifiers using the approach of Donegan et al.[26], as identified from the analyses mentioned above.

We will display forest plots for each meta-analysis with study-specific estimates, confidence intervals and weights, alongside the summary (pooled) meta-analysis estimates and a 95% CI. We will translate our findings to the absolute risk prediction scale to help health professionals tailor treatment decisions to an individual's risk of pre-eclampsia conditional on their covariates

For peer review only - http://bmjopen.bmj.com/site/about/guidelines.xhtml

(prognostic factors) and anticipated treatment effects and any interactions.[27] Penalisation and shrinkage will alleviate overfitting identified using bootstrapping.

Examining potential sources of bias

Small study effects (potential publication bias) will be investigated using funnel plots and test for asymmetry if ten or more studies are in a meta-analysis. To examine the impact of studies where IPD were not shared, we will extract aggregate study-level data (where available) and incorporate them alongside the IPD using the two-stage random effect meta-analysis framework. We will also examine the impact of excluding any trials that are not at low risk of bias.

Dealing with missing variables

A range of strategies will be considered for dealing with missing data in covariates. To analyse randomised trials, mean imputation or the missing indicator method are appropriate to handle missing data in covariates.[28] If necessary, we will use multiple imputations for systematically missing variables (considered plausible), which involves borrowing information across studies while allowing for heterogeneity and clustering in a multi-level imputation model.[29]

Health economic and decision-analytic modelling

Decision model

The cost-effectiveness analysis will be designed and analysed following state of the art methods and analysis in the economic evaluation of healthcare programmes.[30] We will develop a decision tree to determine the cost-effectiveness of calcium supplementation regimens during pregnancy for the prevention of pre-eclampsia. A decision tree is a diagrammatic representation

For peer review only - http://bmjopen.bmj.com/site/about/guidelines.xhtml

of a decision analysis in which chains of choices are identified, each conditional on a prior choice and with outcomes and probabilities[31]. The model structure will be developed based on previous models.[32-36] The results of the cost-effectiveness analysis will be reported according to the 2022 Consolidated Health Economic Evaluation Reporting Standards (CHEERS) statement.[37]

The main outcome of the model will be the incremental cost-effectiveness ratio (ICER). The ICER expresses the additional costs needed to achieve an additional unit of health outcome, i.e., the incremental cost per case of PE/E avoided. Mathematically, ICER can be expressed as:

$$\frac{Cost_1 - Cost_0}{Health\ benefits_1 - Health\ benefits_0}$$

Where 1 represents the intervention group, and 0 represents the comparator group.

Intervention and comparators

The interventions to be evaluated (calcium supplementation regimens), as well as their potential comparators, will be defined according to the parent study's "individual participant data (IPD) meta-analysis".

Target population

The decision model will be applied to a hypothetical population of pregnant women and women of reproductive age who are not yet pregnant but intend to become pregnant, regardless of their risk for pre-eclampsia and their daily calcium intake. Other populations considered will be pregnant women with a high risk of pre-eclampsia and pregnant women with low calcium intake.

For peer review only - http://bmjopen.bmj.com/site/about/guidelines.xhtml

Study perspective

The study will be conducted from the public healthcare system perspective using IPD estimates for Argentina and published literature.

Measurement of effectiveness

The health benefits will be measured as cases of PE/E avoided, life years (LY) gained, and disability-adjusted life years (DALYs) avoided. For women, we will estimate the LY gained subtracting the life expectancy from the mean age of an eclampsia patient, whilst for newborn LY gained will be consider as the average life expectancy in the country. We will use disability weights from the global burden of diseases and country-specific life-expectancy tables for Argentina.[38, 39] Results will be presented as cost per case of PE/E avoided, cost per LY gained and cost per DALYs averted.

Estimating resources and costs

The analysis also will include two main cost categories:

i. Costs of implementing the interventions (calcium acquisition costs, etc.)

ii. Costs associated with using healthcare services by individuals in both the intervention and comparator groups (hospital stay costs in different complexity of care, laboratory tests, among others). The costs of health events will be estimated for both mother and children using the micro-costing method.[40]

Time horizon

For peer review only - http://bmjopen.bmj.com/site/about/guidelines.xhtml

The time horizon will be from pre or early pregnancy until the discharge of mother and child from the hospital.

Discount rate

Since all costs and PE/E cases will occur within the first year, no discounting will be applied to either cost and PE/E cases. For LY and DALYs, a 3% discount rate will be used in accordance with Bill and Melinda Gates Foundation Reference Case guidelines for LMIC.[41]

Currency, date, conversions

The costs of implementing the intervention and those associated with the use of healthcare services by individuals will be valued in local currency and then converted to US dollars using international market exchange rates and international dollars through the purchasing power parity conversion factor published by the World Bank database.[42]

Cost-effectiveness threshold

To define whether the intervention is cost-effective, as the hypothesis is that calcium supplementation will not be "better and cost-saving" than placebo, it will be necessary to establish a decision rule, defined as a willingness-to-pay value for the outcome of interest will be used as a threshold. Despite previous use and recommendations of higher thresholds, such as the World Health Organization's recommendation of up to 3 times the gross domestic product (GDP) per disability-adjusted life-year[43], we will adopt a more stringent threshold consistent with recent studies: 1 times GDP per capita per DALY or QALY.[44, 45] That is, if for a given intervention the ICER lies above this threshold, then it will be deemed too expensive in relation

For peer review only - http://bmjopen.bmj.com/site/about/guidelines.xhtml

to its added benefit and thus not cost-effective, whereas if the ICER lies below this threshold, the intervention will be judged cost-effective and a "good buy". The GDP per capita will be obtained from the World Bank database.[42]

Sensitivity analysis

Sensitivity analysis will be used to report and assess the level of confidence (or uncertainty) that may be associated with the key model parameters (calcium efficacy, etc.). A tornado diagram (deterministic sensitivity analysis) will be generated to plot univariate variations in ICER due to defined variations in key parameters. Probabilistic sensitivity analysis will additionally be performed using 2,000 Monte Carlo simulations. We simultaneously sampled from the distributions of each input parameter in each simulation to estimate the "probability" of the intervention being cost-effective at different thresholds.

DISCUSSION

We propose an IPD meta-analysis of randomised trials to evaluate the effects of calcium supplementation in preventing pre-eclampsia, its complications, and other maternal and fetal-neonatal complications. We will also use an IPD network meta-analysis to compare and rank intervention effects for the various calcium regimens (and doses). In addition, we will assess the cost-effectiveness of calcium supplementation to prevent pre-eclampsia using a model-based economic evaluation for use in LMIC.

The 2018 Guideline Development Groups (GDG) update reported that calcium supplementation is likely to increase equity. Universal calcium supplementation is expected to prevent 21,500

For peer review only

For peer review only - http://bmjopen.bmj.com/site/about/guidelines.xhtml

maternal deaths each year and reduce maternal disability-adjusted life years (DALYs) by 620,000.[46] However, the dose and timing of choice for optimal calcium supplementation to prevent pre-eclampsia are not yet known. With access to IPD containing over 15,000 participants, our IPD meta-analysis will have a larger sample size than any individual study trying to identify if a particular subgroup benefits the most from calcium supplementation and determine the effects on rare but important outcomes of early-onset pre-eclampsia (delivery <34 weeks' gestation), stillbirth and perinatal deaths, and complications such as HELLP syndrome. By accessing the data on the actual timing of commencement of the intervention, the amount of calcium taken by individual women, and their adherence, we can determine if there is an interaction between the effect of calcium treatment and the exact dose taken by the woman. We can then tailor recommendations to the individual conditional on dose and adherence. Furthermore, our IPD meta-analysis will allow us to tailor calcium treatment strategies considering treatment effects on individual-level factors (including prognostic factors and treatment-covariate interactions). We can model prognostic factors to predict a women's pre-eclampsia risk better, conditional on prognostic factors and the expected response to calcium treatment. Thus, we will combine baseline risk and treatment response information to guide treatment decisions based on individual-level information.

The WHO GDG also highlighted an overall lack of information on the cost-effectiveness of calcium supplementation in LMICs, which is crucial to plan implementation. Therefore, we will evaluate the cost-effectiveness of different calcium supplementation strategies in the LMICs context. To facilitate the adoption of the economic model, we will provide the model in an open-

For peer review only - http://bmjopen.bmj.com/site/about/guidelines.xhtml

access format. Other researchers can input their country-specific epidemiological and cost data to determine the cost-effectiveness estimates for their countries.

The findings of this IPD meta-analysis and cost-effectiveness analysis will directly inform guidelines and policymakers in LMICs. The results will assist healthcare managers, other healthcare service providers, and policymakers make informed decisions regarding the ongoing use of calcium or future calcium supplementation strategies to prevent pre-eclampsia based on the efficiency principle.

ETHICS AND DISSEMINATION

The current project involves a meta-analysis of anonymised datasets. No further ethical approvals are needed for this project. Guidance on participant data storage and management will be adhered to. The dataset is not open access. Findings will be published in peer-reviewed journals, presented at UK national and international conferences, shared with policymakers and international organisations, and disseminated to women and their families through links with patient groups and relevant charities.

AUTHORS' CONTRIBUTIONS

ST and JA planned the study, and TR developed the protocol. TR prepared the initial drafts of the manuscript with additional input from JA, AP, JPV, LS, RDR, JH, GC, APB, HM, MAB, TY, ZQ, and GC. TR and JA designed the tables. All authors contributed to the drafts and final version of the manuscript.

For peer review only

FUNDING STATEMENT

The UKRI Medical Research Council supports this work – Global Maternal and Neonatal Health grant number MR/T010185/1. This work is also funded by the UNDP/UNFPA/UNICEF/WHO/World Bank Special Programme of Research, Development and Research Training in Human Reproduction (HRP), Department of Sexual and Reproductive Health and Research (RHR), World Health Organization. JPV is supported by the NHMRC Investigator grant.

COMPETING INTERESTS STATEMENT

None declared.

REFERENCES

1. von Dadelszen P, Magee LA. Preventing deaths due to the hypertensive disorders of pregnancy. Best Pract Res Clin Obstet Gynaecol. 2016;36:83-102. doi: 10.1016/j.bpobgyn.2016.05.005. PubMed PMID: 27531686; PubMed Central PMCID: PMCPMC5096310.
2. Khan KS, Wojdyla D, Say L, Gülmezoglu AM, Van Look PF. WHO analysis of causes of maternal death: a systematic review. Lancet. 2006;367(9516):1066-74. doi: 10.1016/S0140-6736(06)68397-9. PubMed PMID: 16581405.
3. Clark SL, Belfort MA, Dildy GA, Herbst MA, Meyers JA, Hankins GD. Maternal death in the 21st century: causes, prevention, and relationship to cesarean delivery. Am J Obstet Gynecol. 2008;199(1):36.e1-5; discussion 91-2. e7-11. Epub 2008/05/02. doi: 10.1016/j.ajog.2008.03.007. PubMed PMID: 18455140.
4. Organisation WH. Thirteenth General Programme of Work 2019-2023 Online: World Health Organisation; 2019 [cited 2022 23/05/2022]. Available from: https://apps.who.int/iris/bitstream/handle/10665/324775/WHO-PRP-18.1-eng.pdf.
5. Villar J, Belizan JM, Fischer PJ. Epidemiologic observations on the relationship between calcium intake and eclampsia. International Journal of Gynecology and Obstetrics. 1983;21(4):271-8. PubMed PMID: 13008949.
6. Belizan JM, Villar J, Repke J. The relationship between calcium intake and pregnancy-induced hypertension: up-to-date evidence. American Journal of Obstetrics & Gynecology. 1988;158(4):898-902. PubMed PMID: 3284363.

For peer review only - http://bmjopen.bmj.com/site/about/guidelines.xhtml

7. Repke JT, Villar J. Pregnancy-induced hypertension and low birth weight: the role of calcium. American Journal of Clinical Nutrition. 1991;54(1 Suppl):237S-41S. PubMed PMID: 2053568.
8. Cormick G, Betran AP, Romero IB, Lombardo CF, Gulmezoglu AM, Ciapponi A, et al. Global inequities in dietary calcium intake during pregnancy: a systematic review and meta-analysis. 2019;1(4):444-56.
9. Hofmeyr GJ, Lawrie TA, Atallah AN, Torloni MR. Calcium supplementation during pregnancy for preventing hypertensive disorders and related problems. 2018;1:Cd001059.
10. World Health Organization. (2018). Calcium supplementation during pregnancy for prevention of pre-eclampsia and its complications.
11. Kassebaum NJ, Bertozzi-Villa A, Coggeshall MS, Shackelford KA, Steiner C, Heuton KR, et al. Global, regional, and national levels and causes of maternal mortality during 1990-2013: a systematic analysis for the Global Burden of Disease Study 2013. Lancet. 2014;384(9947):980-1004. doi: 10.1016/S0140-6736(14)60696-6. PubMed PMID: 24797575; PubMed Central PMCID: PMCPMC4255481.
12. WHO U, UNFPA, World Bank Group and the United Nations Population Division. Trends in maternal mortality 2000 to 2017: estimates by WHO, UNICEF, UNFPA, World Bank Group and the United Nations Population Division. . 2019.
13. WHO Guidelines Approved by the Guidelines Review Committee. WHO recommendation: Calcium supplementation during pregnancy for the prevention of pre-eclampsia and its complications. Geneva: World Health Organization
© World Health Organization 2018.; 2018.
14. Stewart LA, Clarke M, Rovers M, Riley RD, Simmonds M, Stewart G, et al. Preferred Reporting Items for Systematic Review and Meta-Analyses of individual participant data: the PRISMA-IPD Statement. JAMA. 2015;313(16):1657-65. doi: 10.1001/jama.2015.3656. PubMed PMID: 25919529.
15. WHO recommendation: Calcium supplementation during pregnancy for the prevention of pre-eclampsia and its complications. 2018.
16. Duffy J, Cairns AE, Richards-Doran D, van 't Hooft J, Gale C, Brown M, et al. A core outcome set for pre-eclampsia research: an international consensus development study. BJOG. 2020;127(12):1516-26. Epub 20200621. doi: 10.1111/1471-0528.16319. PubMed PMID: 32416644.
17. Sterne JAC, Savović J, Page MJ, Elbers RG, Blencowe NS, Boutron I, et al. RoB 2: a revised tool for assessing risk of bias in randomised trials. BMJ. 2019;366:l4898. Epub 2019/08/28. doi: 10.1136/bmj.l4898. PubMed PMID: 31462531.
18. Alfirevic Z KF, Stewart F, Jones L, Hampson L, on behalf of Pregnancy and Childbirth Editorial Board. Identifying and handling potentially untrustworthy trials in Pregnancy and Childbirth Cochrane Reviews 2021 [cited 2022 27/05/2022]. Available from: https://pregnancy.cochrane.org/news/identifying-and-handling-potentially-untrustworthy-trials-pregnancy-and-childbirth-cochrane.
19. Demidenko E. Sample size and optimal design for logistic regression with binary interaction. Stat Med. 2008;27(1):36-46. Epub 2007/07/20. doi: 10.1002/sim.2980. PubMed PMID: 17634969.
20. Ensor J, Burke DL, Snell KIE, Hemming K, Riley RD. Simulation-based power calculations for planning a two-stage individual participant data meta-analysis. BMC Med Res

For peer review only - http://bmjopen.bmj.com/site/about/guidelines.xhtml

Methodol. 2018;18(1):41. doi: 10.1186/s12874-018-0492-z. PubMed PMID: 29776399; PubMed Central PMCID: PMCPMC5960205.
21. Burke DL, Ensor J, Riley RD. Meta-analysis using individual participant data: one-stage and two-stage approaches, and why they may differ. Statistics in medicine. 2017;36(5):855-75. doi: 10.1002/sim.7141. PubMed PMID: 27747915; PubMed Central PMCID: PMCPMC5297998.
22. Hua H, Burke DL, Crowther MJ, Ensor J, Tudur Smith C, Riley RD. One-stage individual participant data meta-analysis models: estimation of treatment-covariate interactions must avoid ecological bias by separating out within-trial and across-trial information. Stat Med. 2017;36(5):772-89. doi: 10.1002/sim.7171. PubMed PMID: 27910122; PubMed Central PMCID: PMCPMC5299543.
23. Riley RD, Debray TPA, Fisher D, Hattle M, Marlin N, Hoogland J, et al. Individual participant data meta-analysis to examine interactions between treatment effect and participant-level covariates: Statistical recommendations for conduct and planning. Stat Med. 2020;39(15):2115-37. Epub 2020/04/30. doi: 10.1002/sim.8516. PubMed PMID: 32350891; PubMed Central PMCID: PMCPMC7401032.
24. Riley RD, Jackson D, Salanti G, Burke DL, Price M, Kirkham J, et al. Multivariate and network meta-analysis of multiple outcomes and multiple treatments: rationale, concepts, and examples. BMJ. 2017;358:j3932. Epub 2017/09/15. doi: 10.1136/bmj.j3932. PubMed PMID: 28903924; PubMed Central PMCID: PMCPMC5596393 interests and declare: none.
25. Dias S, Welton NJ, Caldwell DM, Ades AE. Checking consistency in mixed treatment comparison meta-analysis. Stat Med. 2010;29(7-8):932-44. doi: 10.1002/sim.3767. PubMed PMID: 20213715.
26. Donegan S, Williamson P, D'Alessandro U, Garner P, Smith CT. Combining individual patient data and aggregate data in mixed treatment comparison meta-analysis: Individual patient data may be beneficial if only for a subset of trials. Stat Med. 2013;32(6):914-30. Epub 2012/09/17. doi: 10.1002/sim.5584. PubMed PMID: 22987606.
27. Kent DM, Steyerberg E, van Klaveren D. Personalized evidence based medicine: predictive approaches to heterogeneous treatment effects. BMJ. 2018;363:k4245. Epub 2018/12/10. doi: 10.1136/bmj.k4245. PubMed PMID: 30530757; PubMed Central PMCID: PMCPMC6889830.
28. Sullivan TR, White IR, Salter AB, Ryan P, Lee KJ. Should multiple imputation be the method of choice for handling missing data in randomized trials? Stat Methods Med Res. 2018;27(9):2610-26. Epub 2016/12/19. doi: 10.1177/0962280216683570. PubMed PMID: 28034175; PubMed Central PMCID: PMCPMC5393436.
29. Quartagno M, Grund S, Carpenter J. A flexible package for two-level joint modelling multiple imputation. The R Journal. 2019.
30. Drummond MF, Sculpher MJ, Claxton K, Stoddart GL, Torrance GW. Methods for the Economic Evaluation of Health Care Programmes . 4th ed. Oxford: Oxford University Press; 2015.
31. Culyer AJ. The dictionary of health economics. 2nd ed. Chelthenham, UK ;: Edward Elgar; 2010.
32. Meads CA, Cnossen JS, Meher S, Juarez-Garcia A, ter Riet G, Duley L, et al. Methods of prediction and prevention of pre-eclampsia: systematic reviews of accuracy and effectiveness literature with economic modelling. Health technology assessment. 2008;12(6):iii-iv, 1-270. PubMed PMID: 18331705.

For peer review only - http://bmjopen.bmj.com/site/about/guidelines.xhtml

33. Chicaíza-Becerra LA, García-Molina M, Oviedo-Ariza SP, Urrego-Novoa JR, Rincón-Rodríguez CJ, Rubio-Romero JA, et al. [Cost effectiveness of calcium supplement in reducing preeclampsia-related maternal mortality in Colombia]. Rev Salud Publica (Bogota). 2016;18(2):300-10. doi: 10.15446/rsap.v18n2.48776. PubMed PMID: 28453041.
34. Feldhaus I, LeFevre AE, Rai C, Bhattarai J, Russo D, Rawlins B, et al. Optimizing treatment for the prevention of pre-eclampsia/eclampsia in Nepal: is calcium supplementation during pregnancy cost-effective? Cost Eff Resour Alloc. 2016;14:13. Epub 20161228. doi: 10.1186/s12962-016-0062-3. PubMed PMID: 28035193; PubMed Central PMCID: PMCPMC5192578.
35. Meertens LJE, Scheepers HCJ, Willemse J, Spaanderman MEA, Smits LJM. Should women be advised to use calcium supplements during pregnancy? A decision analysis. 2018;1(1).
36. Memirie ST, Tolla MT, Desalegn D, Hailemariam M, Norheim OF, Verguet S, et al. A cost-effectiveness analysis of maternal and neonatal health interventions in Ethiopia. 2019;1(4):289-97.
37. Husereau D, Drummond M, Augustovski F, de Bekker-Grob E, Briggs AH, Carswell C, et al. Consolidated Health Economic Evaluation Reporting Standards 2022 (CHEERS 2022) statement: updated reporting guidance for health economic evaluations. BMJ. 2022;376:e067975. Epub 20220111. doi: 10.1136/bmj-2021-067975. PubMed PMID: 35017145; PubMed Central PMCID: PMCPMC8749494.
38. Mathers CD, Sadana, Ritu , Salomon, Joshua A , Murray, Christopher J. L , Lopez, Alan D. Estimates of DALE for 191 countries: methods and results. World Health Organization: 2000.
39. Health statistics and information systems. Metrics: Disability-Adjusted Life Year (DALY). Quantifying the Burden of Disease from mortality and morbidity. https://www.who.int/healthinfo/global_burden_disease/metrics_daly/en/.
40. Mogyorosy Z, Smith P. The Main Methodological Issues in Costing Health Care Services: A Literature Review.2005. Available from: https://www.york.ac.uk/che/pdf/rp7.pdf.
41. Bill and Melinda Gates Foundation NI, the Health Intervention and Technology Assessment Program (Thailand), and the University of York, Centre for Health Economics. Bill and Melinda Gates Foundation Methods for Economic Evaluation Project (MEEP) [Internet] 2014. Available from: http://www.idsihealth.org/wp-content/uploads/2016/05/Gates-Reference-case-what-it-is-how-to-use-it.pdf.
42. Bank W. Purchasing Power Parities and the Size of World Economies: Results from the 2017 International Comparison Program2020. Available from: https://openknowledge.worldbank.org/handle/10986/33623.
43. Health WCoMa. Macroeconomics and health: investing in health for economic development/report of the Commission on Macroeconomics and Health2001. Available from: https://apps.who.int/iris/handle/10665/42435.
44. Ochalek J, Lomas J, Claxton K. Estimating health opportunity costs in low-income and middle-income countries: a novel approach and evidence from cross-country data. BMJ Glob Health. 2018;3(6):e000964. Epub 20181105. doi: 10.1136/bmjgh-2018-000964. PubMed PMID: 30483412; PubMed Central PMCID: PMCPMC6231096.
45. Woods B, Revill P, Sculpher M, Claxton K. Country-Level Cost-Effectiveness Thresholds: Initial Estimates and the Need for Further Research. Value Health. 2016;19(8):929-

For peer review only - http://bmjopen.bmj.com/site/about/guidelines.xhtml

35. doi: 10.1016/j.jval.2016.02.017. PubMed PMID: 27987642; PubMed Central PMCID: PMCPMC5193154.
46. Bhutta ZA, Ahmed T, Black RE, Cousens S, Dewey K, Giugliani E, et al. What works? Interventions for maternal and child undernutrition and survival. The Lancet. 2008;371(9610):417-40. doi: 10.1016/s0140-6736(07)61693-6.

For peer review only

For peer review only - http://bmjopen.bmj.com/site/about/guidelines.xhtml

BMJ Open

Calcium supplementation to prevent pre-eclampsia: protocol for an individual participant data meta-analysis, network meta-analysis, and health economic evaluation

Journal:	*BMJ Open*
Manuscript ID	bmjopen-2022-065538.R1
Article Type:	Protocol
Date Submitted by the Author:	23-Mar-2023
Complete List of Authors:	Rocha, Thaís; University of Birmingham, WHO Collaborating Centre for Global Women's Health, Institute of Metabolism and Systems Research<br>Allotey, John; University of Birmingham, WHO Collaborating Centre for Global Women's Health, Institute of Metabolism and Systems Research<br>Palacios , Alfredo ; Institute for Clinical Effectiveness and Health Policy, Health Economics<br>Vogel, Joshua; Burnet Institute, Maternal, Child and Adolescent Health Program<br>Smits, Luc; Maastricht University, Department of Epidemiology, Care and Public Health Research Institute<br>Carroli, Guillermo; Centro Rosarino de Estudios Perinatales (CREP)<br>Mistry, Hema; University of Warwick, Warwick Evidence<br>Young, Taryn; Stellenbosch University, Centre for Evidence-based Health Care, Faculty of Medicine and Health Sciences; South African Medical Research Council, South African Cochrane Centre<br>Qureshi , Zahida ; University of Nairobi Department of Obstetrics and Gynecology<br>Cormick, G; Institute for Clinical Effectiveness and Health Policy<br>Snell, Kym IE; Keele University<br>Abalos, E; Centro Rosarino de Estudios Perinatales, Moreno, Argentina<br>Pena-Rosas, Juan-Pablo; World Health Organization, Reproductive Health and Research<br>Khan , Khalid; University of Granada Faculty of Medicine, Public Health<br>Larbi, Koiwah Koi; Action on Preeclampsia (APEC)<br>Thorson, Anna; World Health Organization, Reproductive Health and Research<br>Singata-Madliki, Mandisa; East London Hospital Complex, Effective Care Research Unit (ECRU)<br>Hofmeyr, G Justus ; Frere Hospital, Obstetrics and Gynaecology<br>Bohren, Meghan ; University of Melbourne School of Population and Global Health<br>Riley, Richard; Keele University<br>Betran, Ana Pilar; World Health Organization, Reproductive Health and Research<br>Thangaratinam, Shakila; University of Birmingham College of Medical and Dental Sciences, WHO Collaborating Centre for Global Women's Health, Institute of Metabolism and Systems Research; Birmingham Women's and Children's Hospitals NHS Foundation Trust

**Primary Subject Heading**:	Obstetrics and gynaecology
Secondary Subject Heading:	Epidemiology, Evidence based practice, Global health, Health economics
Keywords:	HEALTH ECONOMICS, Maternal medicine < OBSTETRICS, PUBLIC HEALTH

For peer review only - http://bmjopen.bmj.com/site/about/guidelines.xhtml

Calcium supplementation to prevent pre-eclampsia: protocol for an individual participant data meta-analysis, network meta-analysis, and health economic evaluation

Thaís Rocha,[1] John Allotey,[1] Alfredo Palacios,[2] Joshua P Vogel,[3] Luc Smits,[4] Guillermo Carroli,[5] Hema Mistry,[6] Taryn Young,[7] Zahida Qureshi,[8] Gabriela Cormick,[9] Kym IE Snell,[10] Edgardo Abalos,[5] Juan Pablo Pena-Rosas,[11] Khalid Khan,[12] Koiwah Koi Larbi,[13] Anna Thorson,[11] Mandisa S Madliki,[14] Justus Hofmeyr,[14] Meghan A Bohren,[15] Richard D Riley,[10] Ana Pilar Betrán,[11] Shakila Thangaratinam,[1,16] on behalf of the International Calcium in Pregnancy (i-CIP) Collaborative Network

AFFILIATIONS:

1. WHO Collaborating Centre for Global Women's Health, Institute of Metabolism and Systems Research, University of Birmingham, Birmingham, UK
2. Institute for Clinical Effectiveness and Health Policy (IECS), Buenos Aires, Argentina
3. Maternal, Child and Adolescent Health Program, Burnet Institute, Melbourne, Australia
4. Department of Epidemiology, Care and Public Health Research Institute, Maastricht University, Maastricht, Netherlands
5. Centro Rosarino de Estudios Perinatales (CREP), Rosario, Argentina
6. Clinical Trials Unit, Warwick Medical School, University of Warwick, Coventry, UK
7. Centre for Evidence-based Health Care, Division of Epidemiology and Biostatistics, Department of Global Health, Faculty of Medicine and Health Sciences, Stellenbosch University, Stellenbosch, South Africa
8. The University of Nairobi, Nairobi, Kenya
9. Department of Mother and Child Health Research, Institute for Clinical Effectiveness and Health Policy (IECS-CONICET), Buenos Aires, Argentina

For peer review only - http://bmjopen.bmj.com/site/about/guidelines.xhtml

For peer review only

10. Centre for Prognosis Research, School of Medicine, Keele University, Staffordshire, UK
11. UNDP, UNFPA, UNICEF, WHO, World Bank Special Programme of Research, Development and Research Training in Human Reproduction (HRP), Department of Reproductive Health and Research, World Health Organization, Geneva, Switzerland
12. University of Granada, Granada, Spain
13. Action on Preeclampsia Charity (APEC), Ghana
14. Effective Care Research Unit, University of Witwatersrand, South Africa
15. Gender and Women's Health Unit, Centre for Health Equity, School of Population and Global Health, University of Melbourne, Melbourne, Australia
16. Birmingham Women's and Children's NHS Foundation Trust, Birmingham, UK

CORRESPONDENCE TO:

John Allotey

Institute of Metabolism and Systems Research, University of Birmingham, Birmingham B15 2TT, UK

j.allotey.1@bham.ac.uk

WORD COUNT: 3,646 words

KEYWORDS: pre-eclampsia, calcium supplementation, pregnancy, IPD meta-analysis, economic evaluation, network meta-analysis

For peer review only

For peer review only - http://bmjopen.bmj.com/site/about/guidelines.xhtml

ABSTRACT

Introduction

Low dietary calcium intake is a risk factor for pre-eclampsia, a major contributor to maternal and perinatal mortality and morbidity worldwide. Calcium supplementation can prevent pre-eclampsia in women with low dietary calcium. However, the optimal dose and timing of calcium supplementation are not known. We plan to undertake an individual participant data (IPD) meta-analysis of randomised trials to determine the effects of various calcium supplementation regimens in preventing pre-eclampsia and its complications and rank these by effectiveness. We also aim to evaluate the cost-effectiveness of calcium supplementation to prevent pre-eclampsia.

Methods and analysis

We will identify randomised trials on calcium supplementation before and during pregnancy by searching major electronic databases including Embase, CINAHL, MEDLINE, CENTRAL, PubMed, Scopus, AMED, LILACS, POPLINE, AIM, IMSEAR, ClinicalTrials.gov, and the WHO International Clinical Trials Registry Platform, without language restrictions, from inception to February 2022. Primary researchers of the identified trials will be invited to join the International Calcium in Pregnancy (i-CIP) Collaborative Network and share their IPD. We will check each study's IPD for consistency with the original authors before standardising and harmonising the data. We will perform a series of one- and two-stage IPD random-effect meta-analyses to obtain the summary intervention effects on pre-eclampsia with 95% confidence intervals and summary treatment-covariate interactions (maternal risk status, dietary intake, timing of intervention, daily dose of calcium prescribed, and total intake of calcium). Heterogeneity will be summarised using tau-squared, I-squared and 95% prediction intervals for effect in a new study. Sensitivity analysis to explore robustness of statistical and clinical

For peer review only - http://bmjopen.bmj.com/site/about/guidelines.xhtml
For peer review only

assumptions will be carried out. Minor study effects (potential publication bias) will be investigated using funnel plots. A decision-analytic model for use in low- and middle-income countries will assess the cost-effectiveness of calcium supplementation to prevent pre-eclampsia.

Ethics and dissemination

No ethical approvals are required. We will store the data in a secure repository in an anonymised format. The results will be published in peer-reviewed journals.

PROSPERO registration number

CRD42021231276.

ARTICLE SUMMARY

Strengths and limitations of this study

- The individual participant data (IPD) approach will allow us to explore any differential treatment effect across groups, and model how individual-level covariates (e.g., age, risk of pre-eclampsia) interact with treatment effect within the same trial to explain variability in outcomes.
- By analysing data on the actual amount of calcium taken and adherence to the prescribed regimen, we can explore the doses and frequencies of the clinical benefits of calcium supplementation.
- The health-economic analysis will inform decision-makers on current use or future calcium supplementation strategies to prevent pre-eclampsia based on the efficiency principle.

For peer review only - http://bmjopen.bmj.com/site/about/guidelines.xhtml

- Limitations include potential unavailability of individual participant data, which may limit the number of trials included.

INTRODUCTION

Pre-eclampsia is a pregnancy-specific condition characterised by raised blood pressure and protein in the urine. It is a major cause of maternal and perinatal mortality and morbidity worldwide, contributing to 76,000 maternal and half a million perinatal deaths each year; 99% of these are from low-and-middle-income countries (LMICs).[1-3] Most maternal deaths due to pre-eclampsia are preventable. Prevention of pre-eclampsia and its complications is crucial to achieving the health-related Sustainable Development Goals (SDGs),[4] and the World Health Organization's (WHO) Thirteenth General Programme of Work for universal health coverage.[5]

Low dietary calcium is a recognised risk factor for pre-eclampsia.[6-8] In LMICs, 80% of pregnant women have a mean calcium intake below the population Institute of Medicine recommended level of 800mg/day,[9] compared with low intake in only about a quarter of pregnant women in high-income countries (HIC).[10] Calcium supplementation in pregnancy has been shown to reduce the risk of pre-eclampsia.[11] In populations with low dietary calcium intake and in those at high risk of developing pre-eclampsia, the WHO recommends 1.5–2.0g per day of oral elemental calcium supplementation during pregnancy to reduce the risk of pre-eclampsia, although there is no clear recommendation on the timing of initiation.[12]

A Cochrane review showed that high dose (≥1000mg per day) of calcium supplementation during pregnancy reduced the risk of pre-eclampsia (eight trials, 10,678 women: average RR

For peer review only - http://bmjopen.bmj.com/site/about/guidelines.xhtml

0.36, 95% CI 0.20 to 0.65; I2 = 76%). But the quality was graded low due to significant heterogeneity from variations in the underlying risk of pre-eclampsia.[11] Evidence for a low dose calcium supplement to prevent pre-eclampsia (<1000 mg/day) is limited. [11] Despite countries including calcium in their essential medicines lists, maternal mortality from hypertensive disorders in LMICs remains high.[13, 14] Optimising calcium intake to prevent preeclampsia is a priority area for the WHO.[5, 15] The 2018 WHO Guideline Development Group (GDG) highlighted research on the minimal dose and optimal commencement schedule for calcium supplementation as a high research priority.[15] It is also not known whether calcium supplementation strategies should target high-risk women only or provide calcium supplements to all pregnant and reproductive-aged women, to confer benefits and be cost-effective in preventing pre-eclampsia.

We plan to undertake an individual participant data (IPD) meta-analysis of calcium supplementation to determine the intervention effects on pre-eclampsia and its complications, assess if the effects vary according to maternal and intervention characteristics, and the cost-effectiveness of the different interventions strategies.

Objectives

Our primary objective is to determine the overall, and differential effects of calcium supplementation (according to maternal and intervention characteristics) on pre-eclampsia adjusted for co-interventions and baseline maternal calcium status, using an IPD meta-analysis.

Our secondary objectives are to:

For peer review only - http://bmjopen.bmj.com/site/about/guidelines.xhtml

- Evaluate the effects of calcium supplementation on (i) maternal outcomes such as maternal death, eclampsia, severe maternal morbidity, admission to intensive care unit, Haemolysis, Elevated Liver enzymes, Low Platelets (HELLP) syndrome; and (ii) perinatal outcomes such as stillbirth, perinatal death, neonatal death, preterm birth, low Apgar score, small for gestational age baby, and admission and length of stay in the neonatal intensive care unit.
- Produce a rank order of calcium supplementation regimens by effectiveness.
- Develop a decision-analytic model to determine the cost-effectiveness of different calcium supplementation strategies in an LMIC setting.

METHODS AND ANALYSIS

Our IPD meta-analytical approach will follow existing methodological guidelines and adhere to the PRISMA-IPD reporting statement.[16] The protocol has been registered on the International Prospective Register of Systematic Reviews (PROSPERO; CRD42021231276).

Patient and public involvement

Women with lived experience of pre-eclampsia will be involved with this work throughout and have informed the design, outcome selection and reporting.

Literature search

We will update the search of the 2018 Cochrane review [11] until February 2022 to identify new trials that have been published since the last conducted search. This will include searches in databases such as Embase, CINAHL, MEDLINE, CENTRAL, PubMed, Scopus, AMED,

For peer review only - http://bmjopen.bmj.com/site/about/guidelines.xhtml

LILACS, POPLINE, AIM, IMSEAR, ClinicalTrials.gov, and WHO International Clinical Trials Registry Platform, using search strategies adapted from the original Cochrane search, and will include terms for pregnancy such as 'pregnan*' or 'wom*', combined with terms for calcium 'calcium*' and randomized trials 'random*' or 'allocation' (see online supplemental appendix 1). No language restrictions will be applied.

Eligibility criteria

Any clinical trial with random allocation (individual or cluster) to calcium supplementation (any dose with or without additional supplements or treatments) before or during pregnancy compared with placebo, aspirin, or routine care will be eligible for inclusion. Non-randomised trials and animal studies will be excluded.

Outcome measures

Study outcomes were informed by the WHO recommendation on calcium supplementation during pregnancy to prevent pre-eclampsia and its complications,[17] and the core outcome set for pre-eclampsia research.[18] The primary outcomes are (i) any onset pre-eclampsia and (ii) early-onset pre-eclampsia (diagnosed <34 weeks' gestation). We will use the authors' reported definition of pre-eclampsia. However, suppose the trial IPD reports relevant variables. In that case, we will redefine pre-eclampsia as high blood pressure (defined as systolic blood pressure ≥140mmHg or diastolic blood pressure ≥90mmHg after 20 weeks of pregnancy) with significant proteinuria (defined as urine protein-creatinine ratio ≥30 mg/mmol or ≥2+ on dipstick testing or ≥300 mg/24 hours or ≥500 mg per litre).

For peer review only - http://bmjopen.bmj.com/site/about/guidelines.xhtml

Our secondary outcomes include maternal and offspring complications such as maternal death, eclampsia, severe maternal morbidity (renal, haematological, neurological, hepatic complications), admission to intensive care unit, HELLP syndrome, stillbirth, neonatal death, admission and length of stay in the neonatal intensive care unit, preterm birth, or small for gestational age (Table 1). We will undertake a subgroup analysis to explore whether the intervention effect is modified by (interacts with) maternal risk status, dietary intake, the timing of intervention, the daily dose of calcium prescribed, and total intake of calcium.

For peer review only

Table 1. Structured research question

**Question components**	
Population	Pregnant women and women of reproductive age who are not yet pregnant but intending to become pregnant.
Intervention	Calcium supplementation (with or without additional supplements or treatments)
Outcomes	**Primary outcome**<br>Any onset pre-eclampsia<br>Early-onset pre-eclampsia (<34 weeks' gestation)<br><br>**Secondary outcomes**<br>*Maternal outcomes*<br>Maternal death<br>Eclampsia<br>Severe maternal morbidity (renal, haematological, neurological, hepatic complications)<br>Admission to intensive care unit<br>HELLP syndrome<br><br>*Neonatal outcomes*<br>Stillbirth, neonatal death<br>Apgar score <7 after 5 minutes<br>Admission to the neonatal intensive care unit within 28 days after birth<br>Preterm birth<br>Small for gestational age baby

For peer review only - http://bmjopen.bmj.com/site/about/guidelines.xhtml

Design of included studies	Randomised trials

Study selection

At least two researchers will independently select studies using a two-stage process. They will first screen the titles and abstracts of studies and then assess the full text of selected studies in detail for eligibility. Disagreement will be resolved via discussion with a third researcher. Data extraction will be done in duplicates. At the study level, extracted data will include country, setting, inclusion and exclusion criteria of participants, intervention, control, primary aim, and definition and assessment of the primary outcome.

Establishment of the International Calcium in Pregnancy (i-CIP) Collaborative Network

We will contact primary researchers of identified studies via email and invite them to join the collaborative network and share their IPD. To date, seven collaborators have joined the network and shared access to anonymised individual data of 16,111 women (Table 2). The network is a global effort to bring together researchers, clinicians and epidemiologists (https://www.icipnetwork.com/). A bespoke database will be set up for collaborators to share data. Authors will be allowed to share their data in any format convenient to them. We will consider all variables recorded in the original studies, even those not reported in the publications. Once deposited, the data will be converted to a standardised format, followed by the range and data consistency checking before merging and harmonising.

Table 2. List of trials current in the i-CIP network and trials that have agreed to share data (total n=17,526 individuals)

For peer review only - http://bmjopen.bmj.com/site/about/guidelines.xhtml

Author, Year	Country	Study population risk of PE / Start of intervention	Intervention	Comparator	Sample size	Data already shared with the i-CIP network
Trials currently in iCIP (n=16,111 individuals, 7 trials) [data available already]						
Villar, 2006	Argentina, Egypt, India, Peru, South Africa, Vietnam	High risk, up to 20 weeks' gestation	1,500 mg calcium carbonate	Placebo	8325	Yes
Levine, 1997	United States of America	Low risk, 13-21 weeks' gestation	2,000 mg calcium carbonate	Placebo	4589	Yes
Belizán, 1991	Argentina	Any risk, 20 weeks' gestation	2,000 mg calcium carbonate	Placebo	1194	Yes
Ettinger, 2009	Mexico	Low risk, first trimester	1,200 mg calcium carbonate	Placebo	670	Yes
Goldberg, 2013	Gambia	Any risk, 18-20 weeks' gestation	1,500 mg calcium carbonate	Placebo	662	Yes
Hofmeyr, 2019	Argentina, South Africa, Zimbabwe	High risk, pre-pregnancy and up to 20 weeks' gestation	500 mg calcium carbonate	Placebo	581	Yes
Azami, 2017	Iran	High risk, > 20 weeks' gestation	800mg calcium carbonate	Multivitamin	90	Yes
Trials that agreed to share IPD (n=1,415 individuals, 7 trials) [data expected to be made available to us]						
Omotayo, 2018	Kenya	Low risk, 16-30 gestational weeks	1,500 mg calcium carbonate	1,000 mg calcium carbonate	990	No
Asemi, 2014	Iran	Low risk, 16 weeks' gestation	Multivitamin-mineral with 250 mg calcium	Multivitamin	104	No
Karamali, 2016	Iran	High risk, 24-26 weeks' gestation	1,000 mg calcium carbonate,	Placebo	60	No

For peer review only - http://bmjopen.bmj.com/site/about/guidelines.xhtml

			50,000 IU vitamin D3			
Samimi, 2016	Iran	High risk, 20 weeks' gestation	1,000 mg calcium carbonate, 50,000 IU vitamin D3	Placebo	60	No
Souza, 2014	Brazil	High risk, 20-27 weeks' gestation	2,000 mg calcium carbonate, 100 mg aspirin	Placebo	49	No
Asemi, 2015	Iran	High risk, 27 weeks' gestation	800 mg calcium carbonate, 200 mg magnesium, 8 mg zinc, 400 IU vitamin D3	Placebo	46	No
Asemi, 2016	Iran	Low risk, 25 weeks' gestation	500 mg calcium carbonate, 200 IU vitamin D3	Placebo	46	No

Quality assessment

The quality of the IPD from each study will be assessed independently by two researchers. We will use the revised Cochrane tool for assessing the risk of bias in randomised trials (RoB2)[19] based on published study characteristics and supplement this with information within the IPD. We will consider six items used in the Cochrane risk of bias tool: sequence generation, allocation concealment, blinding, incomplete outcome data, selective outcome reporting, and other potential sources of bias. We will conduct sensitivity analyses to examine the robustness of statistical and clinical conclusions to inform the inclusion or exclusion of trials considered to be at high risk of bias.

Data and integrity checks

For peer review only - http://bmjopen.bmj.com/site/about/guidelines.xhtml
For peer review only

We will perform integrity checks of IPD received for each trial by evaluating the integrity of randomisation and follow-up procedures and reviewing the completeness and accuracy of the data.[20] Any inconsistencies found (missing data, extreme values, discrepancies between the trial report and the data) will be resolved with the original study authors. The study progress and discrepancies will be recorded.

Sample size considerations

Formal sample size calculations are not usually undertaken for meta-analyses. A single trial would need 10,847 participants (80% power, 5% error) to detect the interaction odds ratio (OR) of 0.62 between low and high-risk groups, assuming calcium reduces pre-eclampsia by 20% in a low-risk group by another 30% in the high-risk population.[21] Using power calculations by simulating IPD to match aggregate data (e.g., number of participants, events, covariate distributions)[22] from studies promising their IPD so far (17,526 women) and assuming heterogeneity of 1-8% in the rates of pre-eclampsia in the low-risk group in each trial, we will have over 98% power to detect an interaction OR of 0.62 in our IPD meta-analysis.[22] Even when we additionally assume heterogeneity in the overall effect of calcium in the low-risk group from 0.6 to 0.9, the power will still be 90%, illustrating the large sample size available. We will have similar power for other covariates.

Statistical analysis

Overall effect

We will perform a series of one and two-stage IPD random-effect meta-analyses fitted using either frequentist methods (e.g., restricted maximum likelihood with confidence intervals derived

For peer review only - http://bmjopen.bmj.com/site/about/guidelines.xhtml

For peer review only

using Hartung-Knapp correction) or Bayesian methods (e.g., with vague or empirically derived prior distributions). In the two-stage approach, firstly, the IPD will be analysed separately for each study to obtain relevant aggregate data (e.g., a treatment effect estimates and its confidence interval for each study) for each outcome; secondly, this aggregate data will be combined (pooled) across studies using an appropriate meta-analysis model to produce relevant summary results (e.g., a weighted average of the treatment effect). The alternative one-stage approach analyses the IPD from all studies in a single step, using a statistical model (e.g., a mixed development linear, logistic or Cox regression model) that accounts for the clustering of patients within studies and potential heterogeneity between studies. When the same modelling assumptions and estimation methods are used, one-stage and two-stage approaches are similar.[23] The one-stage approach is preferable when rare events are modelled as a more exact likelihood. However, the two-stage approach allows more familiar meta-analysis techniques and graphs (e.g., forest plots). Therefore, we will perform both one-stage and two-stage methods and compare any differences.[23]

Differential effect by subgroups (treatment-covariate interactions)

For each outcome, we will examine differences in pre-defined subgroups to summarise whether the intervention effect is modified by (interacts with) maternal risk status, dietary intake, the timing of intervention, a daily dose of calcium prescribed, and total intake of calcium; this analysis will utilise only within-study information to avoid ecological bias from across study information. The one-stage analyses will be achieved by centring patient-level covariates by their mean and including the mean as an additional covariate.[24] Non-linear interactions with continuous covariates (e.g., risk status) will be examined using restricted cubic splines.[25]

For peer review only - http://bmjopen.bmj.com/site/about/guidelines.xhtml

IPD network meta-analysis

An IPD network meta-analysis will compare and rank intervention effects for the various regimens (and doses), utilising direct and indirect comparisons whilst adjusting for covariates that modify treatment effects to alleviate any inconsistency in the network.[26] The within-study correlation of multiple intervention effects from the same trial will be accounted for (if necessary). A common between-study variance is assumed for all treatment contrasts in the network. We will produce summary (pooled) effect estimates for each treatment contrast (i.e., each pair of strategies in the network) with 95% confidence intervals (CI) and the borrowing of strength statistics (to reveal the contributions of indirect evidence). Based on the results, the ranking of intervention types will be calculated using resampling methods and quantified by the probabilities of being ranked first, second, and last, together with the mean rank and the Surface Under the Cumulative Ranking curve (SUCRA). The consistency assumption will be examined for each treatment comparison with direct and indirect evidence (seen as a closed-loop within the network plot); this involves estimating the direct and indirect evidence and comparing the two.[27] The consistency assumption will also be examined across the whole network using ‘ design-by-treatment interaction’ models, which allow an overall significance test for inconsistency. If evidence of inconsistency is found, explanations will be sought and resolved by adjusting for covariates that act as effect modifiers using the approach of Donegan et al.[28], as identified from the analyses mentioned above.

We will display forest plots for each meta-analysis with study-specific estimates, confidence intervals and weights, alongside the summary (pooled) meta-analysis estimates and a 95% CI.

For peer review only - http://bmjopen.bmj.com/site/about/guidelines.xhtml

We will translate our findings to the absolute risk prediction scale to help health professionals tailor treatment decisions to an individual's risk of pre-eclampsia conditional on their covariates (prognostic factors) and anticipated treatment effects and any interactions.[29] Penalisation and shrinkage will alleviate overfitting identified using bootstrapping.

Examining potential sources of bias

Small study effects (potential publication bias) will be investigated using funnel plots and test for asymmetry if ten or more studies are in a meta-analysis. To examine the impact of studies where IPD were not shared, we will extract aggregate study-level data (where available) and incorporate them alongside the IPD using the two-stage random effect meta-analysis framework. We will also examine the impact of excluding any trials that are not at low risk of bias.

Dealing with missing variables

A range of strategies will be considered for dealing with missing data in covariates. To analyse randomised trials, mean imputation or the missing indicator method are appropriate to handle missing data in covariates.[30] If necessary, we will use multiple imputations for systematically missing variables (considered plausible), which involves borrowing information across studies while allowing for heterogeneity and clustering in a multi-level imputation model.[31]

Health economic and decision-analytic modelling

Decision model

The cost-effectiveness analysis will be designed and analysed following state of the art methods and analysis in the economic evaluation of healthcare programmes.[32] We will develop a

For peer review only - http://bmjopen.bmj.com/site/about/guidelines.xhtml

decision tree to determine the cost-effectiveness of calcium supplementation regimens during pregnancy for the prevention of pre-eclampsia. A decision tree is a diagrammatic representation of a decision analysis in which chains of choices are identified, each conditional on a prior choice and with outcomes and probabilities[33]. The model structure will be developed based on previous models.[34-38] The results of the cost-effectiveness analysis will be reported according to the 2022 Consolidated Health Economic Evaluation Reporting Standards (CHEERS) statement.[39]

The main outcome of the model will be the incremental cost-effectiveness ratio (ICER). The ICER expresses the additional costs needed to achieve an additional unit of health outcome, i.e., the incremental cost per case of PE/E avoided. Mathematically, ICER can be expressed as:

$$\frac{Cost_1 - Cost_0}{Health\ benefits_1 - Health\ benefits_0}$$

Where 1 represents the intervention group, and 0 represents the comparator group.

Intervention and comparators

The interventions to be evaluated (calcium supplementation regimens), as well as their potential comparators, will be defined according to the parent study's "individual participant data (IPD) meta-analysis".

Target population

The decision model will be applied to a hypothetical population of pregnant women and women of reproductive age who are not yet pregnant but intend to become pregnant, regardless of their

For peer review only - http://bmjopen.bmj.com/site/about/guidelines.xhtml

risk for pre-eclampsia and their daily calcium intake. Other populations considered will be pregnant women with a high risk of pre-eclampsia and pregnant women with low calcium intake.

Study perspective

The study will be conducted from the public healthcare system perspective using IPD estimates for Argentina and published literature.

Measurement of effectiveness

The health benefits will be measured as cases of PE/E avoided, life years (LY) gained, and disability-adjusted life years (DALYs) avoided. For women, we will estimate the LY gained subtracting the life expectancy from the mean age of an eclampsia patient, whilst for newborn LY gained will be consider as the average life expectancy in the country. We will use disability weights from the global burden of diseases and country-specific life-expectancy tables for Argentina.[40, 41] Results will be presented as cost per case of PE/E avoided, cost per LY gained and cost per DALYs averted.

Estimating resources and costs

The analysis also will include two main cost categories:

i. Costs of implementing the interventions (calcium acquisition costs, etc.)

ii. Costs associated with using healthcare services by individuals in both the intervention and comparator groups (hospital stay costs in different complexity of care, laboratory tests, among others). The costs of health events will be estimated for both mother and children using the micro-costing method.[42]

For peer review only - http://bmjopen.bmj.com/site/about/guidelines.xhtml

Time horizon

The time horizon will be from pre or early pregnancy until the discharge of mother and child from the hospital.

Discount rate

Since all costs and PE/E cases will occur within the first year, no discounting will be applied to either cost and PE/E cases. For LY and DALYs, a 3% discount rate will be used in accordance with Bill and Melinda Gates Foundation Reference Case guidelines for LMIC.[43]

Currency, date, conversions

The costs of implementing the intervention and those associated with the use of healthcare services by individuals will be valued in local currency and then converted to US dollars using international market exchange rates and international dollars through the purchasing power parity conversion factor published by the World Bank database.[44]

Cost-effectiveness threshold

To define whether the intervention is cost-effective, as the hypothesis is that calcium supplementation will not be "better and cost-saving" than placebo, it will be necessary to establish a decision rule, defined as a willingness-to-pay value for the outcome of interest will be used as a threshold. Despite previous use and recommendations of higher thresholds, such as the World Health Organization's recommendation of up to 3 times the gross domestic product (GDP) per disability-adjusted life-year[45], we will adopt a more stringent threshold consistent

For peer review only - http://bmjopen.bmj.com/site/about/guidelines.xhtml

with recent studies: 1 times GDP per capita per DALY or QALY.[46, 47] That is, if for a given intervention the ICER lies above this threshold, then it will be deemed too expensive in relation to its added benefit and thus not cost-effective, whereas if the ICER lies below this threshold, the intervention will be judged cost-effective and a "good buy". The GDP per capita will be obtained from the World Bank database.[44]

Sensitivity analysis

Sensitivity analysis will be used to report and assess the level of confidence (or uncertainty) that may be associated with the key model parameters (calcium efficacy, etc.). A tornado diagram (deterministic sensitivity analysis) will be generated to plot univariate variations in ICER due to defined variations in key parameters. Probabilistic sensitivity analysis will additionally be performed using 2,000 Monte Carlo simulations. We simultaneously sampled from the distributions of each input parameter in each simulation to estimate the "probability" of the intervention being cost-effective at different thresholds.

ETHICS AND DISSEMINATION

The current project involves a meta-analysis of anonymised datasets. No ethical approvals are needed for this project. Guidance on participant data storage and management will be adhered to. The dataset is not open access. Findings will be published in peer-reviewed journals, presented at UK national and international conferences, shared with policymakers and international organisations, and disseminated to women and their families through links with patient groups and relevant charities.

For peer review only

DISCUSSION

We propose an IPD meta-analysis of randomised trials to evaluate the effects of calcium supplementation in preventing pre-eclampsia, its complications, and other maternal and fetal-neonatal complications. We will also use an IPD network meta-analysis to compare and rank intervention effects for the various calcium regimens (and doses). In addition, we will assess the cost-effectiveness of calcium supplementation to prevent pre-eclampsia using a model-based economic evaluation for use in LMIC.

The 2018 Guideline Development Groups (GDG) update reported that calcium supplementation is likely to increase equity. Universal calcium supplementation is expected to prevent 21,500 maternal deaths each year and reduce maternal disability-adjusted life years (DALYs) by 620,000.[48] However, the dose and timing of choice for optimal calcium supplementation to prevent pre-eclampsia are not yet known. With access to IPD containing over 15,000 participants, our IPD meta-analysis will have a larger sample size than any individual study trying to identify if a particular subgroup benefits the most from calcium supplementation and determine the effects on rare but important outcomes of early-onset pre-eclampsia (delivery <34 weeks' gestation), stillbirth and perinatal deaths, and complications such as HELLP syndrome. By accessing the data on the actual timing of commencement of the intervention, the amount of calcium taken by individual women, and their adherence, we can determine if there is an interaction between the effect of calcium treatment and the exact dose taken by the woman. We can then tailor recommendations to the individual conditional on dose and adherence.

For peer review only - http://bmjopen.bmj.com/site/about/guidelines.xhtml

Furthermore, our IPD meta-analysis will allow us to tailor calcium treatment strategies considering treatment effects on individual-level factors (including prognostic factors and treatment-covariate interactions). We can model prognostic factors to predict a women's pre-eclampsia risk better, conditional on prognostic factors and the expected response to calcium treatment. Thus, we will combine baseline risk and treatment response information to guide treatment decisions based on individual-level information.

The WHO GDG also highlighted an overall lack of information on the cost-effectiveness of calcium supplementation in LMICs, which is crucial to plan implementation. Therefore, we will evaluate the cost-effectiveness of different calcium supplementation strategies in the LMICs context. To facilitate the adoption of the economic model, we will provide the model in an open-access format. Other researchers can input their country-specific epidemiological and cost data to determine the cost-effectiveness estimates for their countries.

Potential limitations of this study include our inability to obtain IPD from all identified trials due to no contact with original study author, willingness to share raw data or because access to primary data is no longer available. These will be clearly reported as part of our PRISMA flow diagram and a sensitivity analysis to examine the impact of non-IPD studies will be carried out by incorporating these with the IPD studies. There may also be variations in how variables are reported in the shared IPD, which may limit our ability to assess whether the intervention effect is modified by these individual-level covariates. We will minimize the above limitation through robust data cleaning and harmonization procedures..

For peer review only - http://bmjopen.bmj.com/site/about/guidelines.xhtml

The findings of this IPD meta-analysis and cost-effectiveness analysis will directly inform guidelines and policymakers in LMICs. The results will assist healthcare managers, other healthcare service providers, and policymakers make informed decisions regarding the ongoing use of calcium or future calcium supplementation strategies to prevent pre-eclampsia based on the efficiency principle.

CONTRIBUTORS

ST and JA planned the study. TR wrote the initial draft of the protocol manuscript with additional input writing input from AP, JPV, LS, KIES, EA, JPP, KK, KKL, AT, MSM, RDR, JH, GCa, APB, HM, MAB, TY, ZQ, and GCo. TR and JA designed the tables. All authors contributed to the drafts and final version of the manuscript. JA and ST are guarantors. The corresponding author attests that all listed authors meet authorship criteria and that no others meeting the criteria have been omitted.

FUNDING

The UKRI Medical Research Council supports this work – Global Maternal and Neonatal Health grant number MR/T010185/1. This work is also funded by the UNDP/UNFPA/UNICEF/WHO/World Bank Special Programme of Research, Development and Research Training in Human Reproduction (HRP), Department of Sexual and Reproductive Health and Research (SRH), World Health Organization. JPV is supported by the NHMRC Investigator grant.

COMPETING INTERESTS

For peer review only - http://bmjopen.bmj.com/site/about/guidelines.xhtml

None declared.

REFERENCES

1. von Dadelszen P, Magee LA. Preventing deaths due to the hypertensive disorders of pregnancy. Best Pract Res Clin Obstet Gynaecol. 2016;36:83-102. doi: 10.1016/j.bpobgyn.2016.05.005. PubMed PMID: 27531686; PubMed Central PMCID: PMCPMC5096310.
2. Khan KS, Wojdyla D, Say L, Gülmezoglu AM, Van Look PF. WHO analysis of causes of maternal death: a systematic review. Lancet. 2006;367(9516):1066-74. doi: 10.1016/S0140-6736(06)68397-9. PubMed PMID: 16581405.
3. Clark SL, Belfort MA, Dildy GA, Herbst MA, Meyers JA, Hankins GD. Maternal death in the 21st century: causes, prevention, and relationship to cesarean delivery. Am J Obstet Gynecol. 2008;199(1):36.e1-5; discussion 91-2. e7-11. Epub 2008/05/02. doi: 10.1016/j.ajog.2008.03.007. PubMed PMID: 18455140.
4. UNDP What are the Sustainable Development Goals? Goal 3, GOOD HEALTH AND WELL-BEING https://www.undp.org/sustainable-development-goals#good-health © 2022 United Nations Development Programme
5. Organisation WH. Thirteenth General Programme of Work 2019-2023 Online: World Health Organisation; 2019 [cited 2022 23/05/2022]. Available from: https://apps.who.int/iris/bitstream/handle/10665/324775/WHO-PRP-18.1-eng.pdf.
6. Villar J, Belizan JM, Fischer PJ. Epidemiologic observations on the relationship between calcium intake and eclampsia. International Journal of Gynecology and Obstetrics. 1983;21(4):271-8. PubMed PMID: 13008949.
7. Belizan JM, Villar J, Repke J. The relationship between calcium intake and pregnancy-induced hypertension: up-to-date evidence. American Journal of Obstetrics & Gynecology. 1988;158(4):898-902. PubMed PMID: 3284363.
8. Repke JT, Villar J. Pregnancy-induced hypertension and low birth weight: the role of calcium. American Journal of Clinical Nutrition. 1991;54(1 Suppl):237S-41S. PubMed PMID: 2053568.
9. Institute of Medicine of the National Academies. Dietary Reference Intakes for Calcium and Vitamin D. Report brief November 2010. Committee to Review Dietary Reference Intakes for Vitamin D and Calcium. Revised March 2011.
10. Cormick G, Betran AP, Romero IB, Lombardo CF, Gulmezoglu AM, Ciapponi A, et al. Global inequities in dietary calcium intake during pregnancy: a systematic review and meta-analysis. 2019;1(4):444-56.
11. Hofmeyr GJ, Lawrie TA, Atallah AN, Torloni MR. Calcium supplementation during pregnancy for preventing hypertensive disorders and related problems. 2018;1:Cd001059.
12. World Health Organization. (2018). Calcium supplementation during pregnancy for prevention of pre-eclampsia and its complications.
13. Kassebaum NJ, Bertozzi-Villa A, Coggeshall MS, Shackelford KA, Steiner C, Heuton KR, et al. Global, regional, and national levels and causes of maternal mortality during 1990-2013: a systematic analysis for the Global Burden of Disease Study 2013. Lancet.

For peer review only - http://bmjopen.bmj.com/site/about/guidelines.xhtml

2014;384(9947):980-1004. doi: 10.1016/S0140-6736(14)60696-6. PubMed PMID: 24797575; PubMed Central PMCID: PMCPMC4255481.
14. WHO U, UNFPA, World Bank Group and the United Nations Population Division. Trends in maternal mortality 2000 to 2017: estimates by WHO, UNICEF, UNFPA, World Bank Group and the United Nations Population Division. 2019.
15. WHO Guidelines Approved by the Guidelines Review Committee. WHO recommendation: Calcium supplementation during pregnancy for the prevention of pre-eclampsia and its complications. Geneva: World Health Organization, 2018.
16. Stewart LA, Clarke M, Rovers M, Riley RD, Simmonds M, Stewart G, et al. Preferred Reporting Items for Systematic Review and Meta-Analyses of individual participant data: the PRISMA-IPD Statement. JAMA. 2015;313(16):1657-65. doi: 10.1001/jama.2015.3656. PubMed PMID: 25919529.
17. WHO recommendation: Calcium supplementation during pregnancy for the prevention of pre-eclampsia and its complications. 2018.
18. Duffy J, Cairns AE, Richards-Doran D, van 't Hooft J, Gale C, Brown M, et al. A core outcome set for pre-eclampsia research: an international consensus development study. BJOG. 2020;127(12):1516-26. Epub 20200621. doi: 10.1111/1471-0528.16319. PubMed PMID: 32416644.
19. Sterne JAC, Savović J, Page MJ, Elbers RG, Blencowe NS, Boutron I, et al. RoB 2: a revised tool for assessing risk of bias in randomised trials. BMJ. 2019;366:l4898. Epub 2019/08/28. doi: 10.1136/bmj.l4898. PubMed PMID: 31462531.
20. Alfirevic Z KF, Stewart F, Jones L, Hampson L, on behalf of Pregnancy and Childbirth Editorial Board. Identifying and handling potentially untrustworthy trials in Pregnancy and Childbirth Cochrane Reviews 2021 [cited 2022 27/05/2022]. Available from: https://pregnancy.cochrane.org/news/identifying-and-handling-potentially-untrustworthy-trials-pregnancy-and-childbirth-cochrane.
21. Demidenko E. Sample size and optimal design for logistic regression with binary interaction. Stat Med. 2008;27(1):36-46. Epub 2007/07/20. doi: 10.1002/sim.2980. PubMed PMID: 17634969.
22. Ensor J, Burke DL, Snell KIE, Hemming K, Riley RD. Simulation-based power calculations for planning a two-stage individual participant data meta-analysis. BMC Med Res Methodol. 2018;18(1):41. doi: 10.1186/s12874-018-0492-z. PubMed PMID: 29776399; PubMed Central PMCID: PMCPMC5960205.
23. Burke DL, Ensor J, Riley RD. Meta-analysis using individual participant data: one-stage and two-stage approaches, and why they may differ. Statistics in medicine. 2017;36(5):855-75. doi: 10.1002/sim.7141. PubMed PMID: 27747915; PubMed Central PMCID: PMCPMC5297998.
24. Hua H, Burke DL, Crowther MJ, Ensor J, Tudur Smith C, Riley RD. One-stage individual participant data meta-analysis models: estimation of treatment-covariate interactions must avoid ecological bias by separating out within-trial and across-trial information. Stat Med. 2017;36(5):772-89. doi: 10.1002/sim.7171. PubMed PMID: 27910122; PubMed Central PMCID: PMCPMC5299543.
25. Riley RD, Debray TPA, Fisher D, Hattle M, Marlin N, Hoogland J, et al. Individual participant data meta-analysis to examine interactions between treatment effect and participant-level covariates: Statistical recommendations for conduct and planning. Stat Med.

For peer review only

For peer review only - http://bmjopen.bmj.com/site/about/guidelines.xhtml

2020;39(15):2115-37. Epub 2020/04/30. doi: 10.1002/sim.8516. PubMed PMID: 32350891; PubMed Central PMCID: PMCPMC7401032.
26. Riley RD, Jackson D, Salanti G, Burke DL, Price M, Kirkham J, et al. Multivariate and network meta-analysis of multiple outcomes and multiple treatments: rationale, concepts, and examples. BMJ. 2017;358:j3932. Epub 2017/09/15. doi: 10.1136/bmj.j3932. PubMed PMID: 28903924; PubMed Central PMCID: PMCPMC5596393 interests and declare: none.
27. Dias S, Welton NJ, Caldwell DM, Ades AE. Checking consistency in mixed treatment comparison meta-analysis. Stat Med. 2010;29(7-8):932-44. doi: 10.1002/sim.3767. PubMed PMID: 20213715.
28. Donegan S, Williamson P, D'Alessandro U, Garner P, Smith CT. Combining individual patient data and aggregate data in mixed treatment comparison meta-analysis: Individual patient data may be beneficial if only for a subset of trials. Stat Med. 2013;32(6):914-30. Epub 2012/09/17. doi: 10.1002/sim.5584. PubMed PMID: 22987606.
29. Kent DM, Steyerberg E, van Klaveren D. Personalized evidence based medicine: predictive approaches to heterogeneous treatment effects. BMJ. 2018;363:k4245. Epub 2018/12/10. doi: 10.1136/bmj.k4245. PubMed PMID: 30530757; PubMed Central PMCID: PMCPMC6889830.
30. Sullivan TR, White IR, Salter AB, Ryan P, Lee KJ. Should multiple imputation be the method of choice for handling missing data in randomized trials? Stat Methods Med Res. 2018;27(9):2610-26. Epub 2016/12/19. doi: 10.1177/0962280216683570. PubMed PMID: 28034175; PubMed Central PMCID: PMCPMC5393436.
31. Quartagno M, Grund S, Carpenter J. A flexible package for two-level joint modelling multiple imputation. The R Journal. 2019.
32. Drummond MF, Sculpher MJ, Claxton K, Stoddart GL, Torrance GW. Methods for the Economic Evaluation of Health Care Programmes. 4th ed. Oxford: Oxford University Press; 2015.
33. Culyer AJ. The dictionary of health economics. 2nd ed. Chelthenham, UK ;: Edward Elgar; 2010.
34. Meads CA, Cnossen JS, Meher S, Juarez-Garcia A, ter Riet G, Duley L, et al. Methods of prediction and prevention of pre-eclampsia: systematic reviews of accuracy and effectiveness literature with economic modelling. Health technology assessment. 2008;12(6):iii-iv, 1-270. PubMed PMID: 18331705.
35. Chicaíza-Becerra LA, García-Molina M, Oviedo-Ariza SP, Urrego-Novoa JR, Rincón-Rodríguez CJ, Rubio-Romero JA, et al. [Cost effectiveness of calcium supplement in reducing preeclampsia-related maternal mortality in Colombia]. Rev Salud Publica (Bogota). 2016;18(2):300-10. doi: 10.15446/rsap.v18n2.48776. PubMed PMID: 28453041.
36. Feldhaus I, LeFevre AE, Rai C, Bhattarai J, Russo D, Rawlins B, et al. Optimizing treatment for the prevention of pre-eclampsia/eclampsia in Nepal: is calcium supplementation during pregnancy cost-effective? Cost Eff Resour Alloc. 2016;14:13. Epub 20161228. doi: 10.1186/s12962-016-0062-3. PubMed PMID: 28035193; PubMed Central PMCID: PMCPMC5192578.
37. Meertens LJE, Scheepers HCJ, Willemse J, Spaanderman MEA, Smits LJM. Should women be advised to use calcium supplements during pregnancy? A decision analysis. 2018;1(1).

For peer review only - http://bmjopen.bmj.com/site/about/guidelines.xhtml

38. Memirie ST, Tolla MT, Desalegn D, Hailemariam M, Norheim OF, Verguet S, et al. A cost-effectiveness analysis of maternal and neonatal health interventions in Ethiopia. 2019;1(4):289-97.
39. Husereau D, Drummond M, Augustovski F, de Bekker-Grob E, Briggs AH, Carswell C, et al. Consolidated Health Economic Evaluation Reporting Standards 2022 (CHEERS 2022) statement: updated reporting guidance for health economic evaluations. BMJ. 2022;376:e067975. Epub 20220111. doi: 10.1136/bmj-2021-067975. PubMed PMID: 35017145; PubMed Central PMCID: PMCPMC8749494.
40. Mathers CD, Sadana R, Salomon JA, Murray CJL, Lopez AD. Estimates of DALE for 191 countries: methods and results. World Health Organization: 2000.
41. Health statistics and information systems. Metrics: Disability-Adjusted Life Year (DALY). Quantifying the Burden of Disease from mortality and morbidity. https://www.who.int/healthinfo/global_burden_disease/metrics_daly/en/.
42. Mogyorosy Z, Smith P. The Main Methodological Issues in Costing Health Care Services: A Literature Review.2005. Available from: https://www.york.ac.uk/che/pdf/rp7.pdf.
43. Bill and Melinda Gates Foundation NI, the Health Intervention and Technology Assessment Program (Thailand), and the University of York, Centre for Health Economics. Bill and Melinda Gates Foundation Methods for Economic Evaluation Project (MEEP) [Internet] 2014. Available from: http://www.idsihealth.org/wp-content/uploads/2016/05/Gates-Reference-case-what-it-is-how-to-use-it.pdf.
44. World Bank. Purchasing Power Parities and the Size of World Economies: Results from the 2017 International Comparison Program2020. Available from: https://openknowledge.worldbank.org/handle/10986/33623.
45. Health WCoMa. Macroeconomics and health: investing in health for economic development/report of the Commission on Macroeconomics and Health2001. Available from: https://apps.who.int/iris/handle/10665/42435.
46. Ochalek J, Lomas J, Claxton K. Estimating health opportunity costs in low-income and middle-income countries: a novel approach and evidence from cross-country data. BMJ Glob Health. 2018;3(6):e000964. Epub 20181105. doi: 10.1136/bmjgh-2018-000964. PubMed PMID: 30483412; PubMed Central PMCID: PMCPMC6231096.
47. Woods B, Revill P, Sculpher M, Claxton K. Country-Level Cost-Effectiveness Thresholds: Initial Estimates and the Need for Further Research. Value Health. 2016;19(8):929-35. doi: 10.1016/j.jval.2016.02.017. PubMed PMID: 27987642; PubMed Central PMCID: PMCPMC5193154.
48. Bhutta ZA, Ahmed T, Black RE, Cousens S, Dewey K, Giugliani E, et al. What works? Interventions for maternal and child undernutrition and survival. The Lancet. 2008;371(9610):417-40. doi: 10.1016/s0140-6736(07)61693-6.

For peer review only

Appendix: Details of search strategies used in IPD meta-analysis on calcium supplementation to prevent pre-eclampsia in low- income and middle-income countries

1. Ovid MEDLINE

1 exp Pregnancy/
2 Pregnant Women/
3 (pregnan$ or gestation$ or antenatal$ or ante-natal$ or prenatal$ or pre-natal$ or post-conception$ or postconception$).ti,ab,kf.
4 or/1-3
5 Calcium/
6 Calcium, Dietary/
7 calcium.ti,ab,kf.
8 or/5-7
9 4 and 8
10 randomized controlled trial.pt.
11 controlled clinical trial.pt.
12 randomized.ab.
13 placebo.ab.
14 Clinical Trials as Topic/
15 randomly.ab.
16 trial.ti.
17 or/10-16
18 9 and 17
19 exp Animals/ not Humans/
20 18 not 19
21 (news or editorial or case reports).pt. or case report.ti.
22 20 not 21
23 remove duplicates from 22

2. Cochrane Central Register of Controlled Trials

#1 [mh Pregnancy]

#2 [mh ^"Pregnant Women"]

#3 (pregnan* or wom* or gestation* or antenatal* or prenatal* or postconception* or ante NEXT natal* or pre NEXT natal* or post NEXT conception*)

#4 #1 OR #2 OR #3

#5 [mh ^"Calcium, Dietary"]

#6 [mh ^calcium]

#7 calcium

#8 #5 OR #6 OR #7

#9 #4 AND #8 in Trials

For peer review only - http://bmjopen.bmj.com/site/about/guidelines.xhtml
For peer review only

3. WHO International Clinical Trials Platform

pregnancy AND calcium OR wom* AND calcium OR gestation* AND calcium OR antenatal* AND calcium OR prenatal* AND calcium OR postconception* AND calcium OR antenatal* AND calcium OR prenatal* AND calcium OR postconception* AND calcium

4. ClinicalTrials.gov

calcium | Interventional Studies | pregnancy OR pregnant OR woman OR women OR gestation OR antenatal OR prenatal OR postconception OR ante-natal OR pre-natal OR post-conception | Studies with Female Participants

5. SCOPUS

((TITLE-ABS-KEY (pregnan* OR wom* OR gestation* OR antenatal* OR "ante-natal*" OR prenatal OR "pre-natal*" OR "post-conception*" OR postconception*) AND TITLE-ABS-KEY (calcium) AND TITLE-ABS-KEY (trial* OR random* OR "clinical stud*" OR "controlled stud*"))) AND NOT ((KEY (animal*) OR TITLE (rat OR rats OR mice OR mouse OR hamster OR hamsters OR bovine OR sheep OR dog OR dogs OR cat OR cats OR rabbit OR rabbits OR calf OR calves OR cow OR cows OR pig OR pigs OR swine OR porcine)) AND NOT KEY (human*)) AND NOT INDEX (medline)

6. CINAHL

S1 (MH "Pregnancy+”)
S2 (MH “Expectant Mothers”)
S3 TI (pregnan* or gestation* or antenatal* or prenatal* or postconception* or ante W3 natal* or pre W3 natal* or post W3 conception*) OR AB (pregnan* or gestation* or antenatal* or prenatal* or postconception* or ante W3 natal* or pre W3 natal* or post W3 conception*)
S4 S1 OR S2 OR S3
S5 (MH "Calcium")
S6 (MH "Calcium, Dietary")
S7 TI calcium OR AB calcium
S8 S5 OR S6 OR S7
S9 S4 AND S8
S10 (MH “randomized controlled trials”)
S11 (MH double-blind studies)
S12 (MH “single-blind studies”)
S13 (MH “random assignment”)
S14 (MH “pretest-posttest design”)
S15 (MH “cluster sample”)
S16 TI (randomised OR randomized)
S17 AB (random*)

For peer review only - http://bmjopen.bmj.com/site/about/guidelines.xhtml

S18 TI (trial)
S19 MH (“sample size”) AND AB (assigned OR allocated OR control)
S20 MH (placebos)
S21 PT (“randomized controlled trial”)
S22 AB (control W5 group)
S23 MH (crossover design) OR MH (comparative studies)
S24 AB (cluster W3 RCT)
S25 S10 OR S11 OR S12 OR S13 OR S14 OR S15 OR S16 OR S17 OR S18 OR S19 OR S20 OR S21 OR S22 OR S23 OR S24
S26 S9 AND S25
S27 (MH Animals+) OR (MH “Animal Studies”) OR TI (animal model*)
S28 MH (Human)
S29 S27 NOT S28
S30 S26 NOT S29

7. PubMed

"(((calcium supplement*) OR (“calcium carbonate”) OR (“calcium gluconate”) OR (“calcium acetate”) OR (“calcium citrate”) OR (“calcium lactate”) OR (“calcium”)) AND ((“Pregnant Women”[Mesh]) OR (“Pregnancy”[Mesh]) OR (“pregnancy”) OR (“pregnant”) OR (“pregnancies”)) AND ((random) OR (randomised) OR (randomized)) AND (trial)"

8. EMBASE, CINHAL, AMED, and LILACS.

"(calcium) AND (pregnan*) AND ((random) OR (randomised) OR (randomized)) AND (trial)”

For peer review only

PRISMA-P (Preferred Reporting Items for Systematic review and Meta-Analysis Protocols) 2015 checklist: recommended items to address in a systematic review protocol*

Section and topic	Item No	Checklist item	Page
**ADMINISTRATIVE INFORMATION**			
Title:			
Identification	1a	Identify the report as a protocol of a systematic review	1
Update	1b	If the protocol is for an update of a previous systematic review, identify as such	NA
Registration	2	If registered, provide the name of the registry (such as PROSPERO) and registration number	3
Authors:			
Contact	3a	Provide name, institutional affiliation, e-mail address of all protocol authors; provide physical mailing address of corresponding author	1-2
Contributions	3b	Describe contributions of protocol authors and identify the guarantor of the review	22
Amendments	4	If the protocol represents an amendment of a previously completed or published protocol, identify as such and list changes; otherwise, state plan for documenting important protocol amendments	NA
Support:			
Sources	5a	Indicate sources of financial or other support for the review	22
Sponsor	5b	Provide name for the review funder and/or sponsor	22
Role of sponsor or funder	5c	Describe roles of funder(s), sponsor(s), and/or institution(s), if any, in developing the protocol	22
**INTRODUCTION**			
Rationale	6	Describe the rationale for the review in the context of what is already known	5
Objectives	7	Provide an explicit statement of the question(s) the review will address with reference to participants, interventions, comparators, and outcomes (PICO)	9
**METHODS**			
Eligibility criteria	8	Specify the study characteristics (such as PICO, study design, setting, time frame) and report characteristics (such as years considered, language, publication status) to be used as criteria for eligibility for the review	8
Information sources	9	Describe all intended information sources (such as electronic databases, contact with study authors, trial registers or other grey literature sources) with planned dates of coverage	7
Search strategy	10	Present draft of search strategy to be used for at least one electronic database, including planned limits, such that it could be repeated	App
Study records:			
Data	11a	Describe the mechanism(s) that will be used to manage records and data throughout the review	10

For peer review only

management			
Selection process	11b	State the process that will be used for selecting studies (such as two independent reviewers) through each phase of the review (that is, screening, eligibility and inclusion in meta-analysis)	9-10
Data collection process	11c	Describe planned method of extracting data from reports (such as piloting forms, done independently, in duplicate), any processes for obtaining and confirming data from investigators	9-12
Data items	12	List and define all variables for which data will be sought (such as PICO items, funding sources), any pre-planned data assumptions and simplifications	10
Outcomes and prioritization	13	List and define all outcomes for which data will be sought, including prioritization of main and additional outcomes, with rationale	8
Risk of bias in individual studies	14	Describe anticipated methods for assessing risk of bias of individual studies, including whether this will be done at the outcome or study level, or both; state how this information will be used in data synthesis	12
Data synthesis	15a	Describe criteria under which study data will be quantitatively synthesised	13-14
	15b	If data are appropriate for quantitative synthesis, describe planned summary measures, methods of handling data and methods of combining data from studies, including any planned exploration of consistency (such as I^2, Kendall's τ)	13-14
	15c	Describe any proposed additional analyses (such as sensitivity or subgroup analyses, meta-regression)	14
	15d	If quantitative synthesis is not appropriate, describe the type of summary planned	NA
Meta-bias(es)	16	Specify any planned assessment of meta-bias(es) (such as publication bias across studies, selective reporting within studies)	16
Confidence in cumulative evidence	17	Describe how the strength of the body of evidence will be assessed (such as GRADE)	NA

*** It is strongly recommended that this checklist be read in conjunction with the PRISMA-P Explanation and Elaboration (cite when available) for important clarification on the items. Amendments to a review protocol should be tracked and dated. The copyright for PRISMA-P (including checklist) is held by the PRISMA-P Group and is distributed under a Creative Commons Attribution Licence 4.0.**

From: Shamseer L, Moher D, Clarke M, Ghersi D, Liberati A, Petticrew M, Shekelle P, Stewart L, PRISMA-P Group. Preferred reporting items for systematic review and meta-analysis protocols (PRISMA-P) 2015: elaboration and explanation. BMJ. 2015 Jan 2;349(jan02 1):g7647.

For peer review only
